# Complex societies and the growth of the law

**DOI:** 10.1038/s41598-020-73623-x

**Published:** 2020-10-30

**Authors:** Daniel Martin Katz, Corinna Coupette, Janis Beckedorf, Dirk Hartung

**Affiliations:** 1 Illinois Tech – Chicago Kent College of Law, Chicago, USA; 2 CodeX – The Stanford Center for Legal Informatics, Stanford, USA; 3 Max Planck Institute for Informatics, Saarbrücken, Germany; 4 Faculty of Law, Ruprecht-Karls-Universität Heidelberg, Heidelberg, Germany; 5 Bucerius Law School, Hamburg, Germany

**Keywords:** Complex networks, Computational science

## Abstract

While many informal factors influence how people interact, modern societies rely upon law as a primary mechanism to formally control human behaviour. How legal rules impact societal development depends on the interplay between two types of actors: the people who create the rules and the people to which the rules potentially apply. We hypothesise that an increasingly diverse and interconnected society might create increasingly diverse and interconnected rules, and assert that legal networks provide a useful lens through which to observe the interaction between law and society. To evaluate these propositions, we present a novel and generalizable model of statutory materials as multidimensional, time-evolving document networks. Applying this model to the federal legislation of the United States and Germany, we find impressive expansion in the size and complexity of laws over the past two and a half decades. We investigate the sources of this development using methods from network science and natural language processing. To allow for cross-country comparisons over time, based on the explicit cross-references between legal rules, we algorithmically reorganise the legislative materials of the United States and Germany into cluster families that reflect legal topics. This reorganisation reveals that the main driver behind the growth of the law in both jurisdictions is the expansion of the welfare state, backed by an expansion of the tax state. Hence, our findings highlight the power of document network analysis for understanding the evolution of law and its relationship with society.

## Introduction

Modern societies rely upon law as the primary mechanism to control their development and manage their conflicts. Through carefully designed rights and responsibilities, institutions and procedures, law can enable humans to engage in increasingly complex social and economic activities. Therefore, law plays an important role in understanding how societies change. To explore the interplay between law and society, we need to study how both co-evolve over time. This requires a firm quantitative grasp of the changes occurring in both domains. But while quantifying societal change has been the subject of tremendous research efforts in fields such as sociology, economics, or social physics for many years^1–6^, much less work has been done to quantify legal change. In fact, legal scholars have traditionally regarded the law as hardly quantifiable, and although there is no dearth of empirical legal studies^7–9^, it is only recently that researchers have begun to apply data science methods to law^10–13^. To date, there have been relatively few quantitative works that explicitly address legal change^14–19^, and almost no scholarship exists that analyses the time-evolving outputs of the legislative and executive branches of national governments at scale. Unlocking these data sources for the interdisciplinary scientific community will be crucial for understanding how law and society interact.

Our work takes a step towards this goal. As a starting point, we hypothesise that an increasingly diverse and interconnected society might create increasingly diverse and interconnected rules. Lawmakers create, modify, and delete legal rules to achieve particular behavioural outcomes, often in an effort to respond to perceived changes in societal needs. While earlier large-scale quantitative work focused on analysing an individual snapshot of laws enacted by national parliaments^20,21^, collections of snapshots offer a window into the dynamic interaction between law and society. Such collections represent complete, time-evolving populations of statutes at the national level. Hence, no sampling is needed for their analysis, and all changes we observe are direct consequences of legislative activity. This feature makes collections of nation-level statutes particularly suitable for investigating temporal dynamics.

To preserve the intended multidimensionality of legal document collections and explore how they change over time, legislative corpora should be modelled as dynamic document networks^20–26^. In particular, since legal documents are carefully organised and interlinked, their structure provides a more direct window into their content and dynamics than their language: Networks honour the deliberate design decisions made by the document authors and circumvent some of the ambiguity problems that natural language-based approaches inherently face. In this paper, we therefore develop an informed data model for legislative corpora, capturing the richness of legislative data for exploration by social physics. We leverage our data model to analyse the evolution of federal statutes in the United States and Germany. Here, we find extensive growth in legal complexity as a function of volume, interconnectivity, and hierarchical structure of the legislation in both countries—evidence that the highly industrialised countries we study seek to manage behaviour by building increasingly complex bodies of legal rules. Searching for the sources of the growth we observe, we draw on graph clustering techniques to locate those legal topics that contribute most to the complexity increase and trace their development over time. Deriving our information on the content of legislative documents directly from the conscious structural choices made by their drafters, we find that the main driver behind the growth of the law in both the United States and Germany is the expansion of the welfare state, backed by an expansion of the tax state. Beyond this high-level picture, our methodology also enables more fine-grained discoveries—for example, we find that during our observation period, the regulation of natural resources in the United States shifted from exploitation to conservation. Thus, we achieve results that would be hard (or even impossible) to obtain using approaches that leverage only the natural language of legislative documents while keeping the amount of subjective judgements to a minimum. Our work highlights the potential of legal network data and document network analysis for studying the interaction between law and society when viewed through the lens of Complex Adaptive Systems (CAS)^17,27–31^, and it opens novel research avenues to the interdisciplinary scientific community.

## Results

### Dynamic network model of legislation

We model 25 years of statutory materials from two advanced industrial countries, the United States and Germany, as time-evolving document networks. To build our original datasets, for the United States, we collect annual snapshots of the United States Code (US Code) from 1994 to 2018 from the Office of the Law Revision Counsel of the U.S. House of Representatives. For Germany, we create a parallel set of yearly snapshots for all federal statutory laws in effect at the beginning of the year in question based on documents from Germany’s primary legal data provider, *juris GmbH*. For details on our data sources, see Sect. 1 of the Supplementary Information (SI).

Each individual law or section (that has not been repealed) contains at least some text, and it may contain nested subsections as well as ingoing and outgoing references. For each country and yearly snapshot, we construct a network of all federal statutes. The entities in this network are the structural elements of the statutes we collect, some of which contain text (i.e., the stipulation of a legal rule). These entities are interconnected by inclusion relationships (e.g., a section containing several paragraphs) and cross-references (i.e., the text of an element referencing another element), and they can be sequentially ordered by their labels. Notably, only one level of the inclusion hierarchy in legislative corpora is uniquely sequentially labelled (this is the Section level in the United States and the § or Article level in Germany). We refer to the structural elements in this layer as *seqitems*. For excerpts from United States law and German law that illustrate their inherent structure, see Sect. 1.3 of the SI.

**Figure 1:**
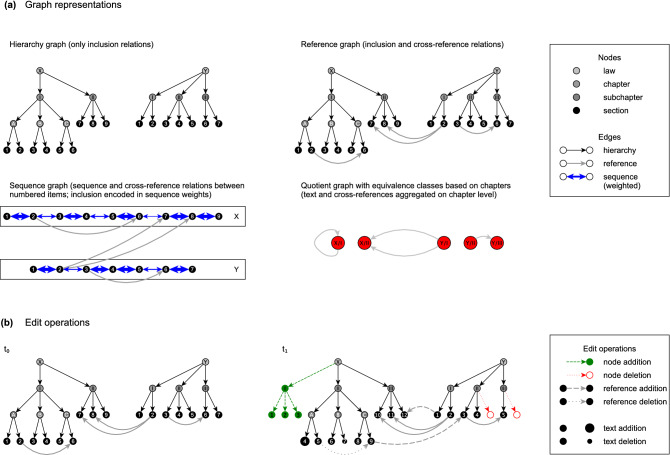
Dynamic network data model for legislative document collections. All figures created by the authors.

In the legislative process, the structure of legislative texts is controlled by the administrative officials drafting the rules. Therefore, *hierarchy*, *reference*, and *sequence* within a corpus of legislative documents contain information about the content of the corpus that is less noisy and easier to parse than its language. To unlock this information for large-scale comparative and dynamic analysis, we model a body of legislation at a certain point in time as a document collection following the Document Object Model (DOM) standard^32^ (for our domain-specific XML Schema Definition [XSD], see Sect. 2.4 of the SI). With each document collection, we associate four graphs as depicted in Fig. 1a, whose formal definitions are given in "Modelling legislative document collections". Our simplest graph is the *hierarchy graph*, which models the inclusion relationships between the structural elements of legal texts. It is a subgraph of the *reference graph*, which models inclusion and cross-reference relationships. From a network science perspective, the reference graph is perhaps the most intuitive representation of a legislative document collection, and all of our other graphs can be derived from it. The *sequence graph* contains only the *seqitems* from the reference graph, which are connected by cross-reference edges and bidirectional *sequence edges* ("Modelling legislative document collections" introduces a parametrized definition of this graph for greater analytical flexibility). The cross-reference edges are unweighted, while the sequence edges have weights proportional to the distance between their endpoints in the undirected version of the hierarchy graph (e.g., a sequence edge between Section $i-1$ and Section *i* in Chapter *A* weighs more than a sequence edge between Section *i* in Chapter *A* and Section $i+1$ in Chapter *B*). The sequence graph expresses how legal practitioners work with a legal text (i.e., they approach a topic through one particular rule, scan its vicinity as long as it is also hierarchically close, possibly follow a cross-reference, then scan the hierarchically close vicinity of a referenced rule). Finally, we define *quotient graphs* based on attributes attached to the elements of our reference graphs. In these graphs, all elements with the same attribute value(s) (e.g., all *seqitems* belonging to the same Chapter) are collapsed into one node, and edges are rerouted accordingly.

The graphs sketched above allow us to compare legislative document collections both *horizontally* (i.e., across nations) and *vertically* (i.e., across time). In particular, hierarchy graphs and reference graphs let us track basic statistics over time (cf. "Assessing legislative growth"), which change when lawmakers add, update, or delete legal rules as depicted in Fig. 1b. Sequence graphs help us align basic elements of legal texts across years (cf. "Comparing document networks over space and time”). Along with quotient graphs, they also facilitate the reorganisation of legislative materials via graph clustering (cf. "Comparing document networks over space and time"), where they allow us to focus on different topics or levels of granularity depending on the research question to be investigated. To the best of our knowledge, there exists no comparably flexible explicit model for legislative document collections in the document network analysis literature. Since we do not use all features of the model in our analysis, exploiting the power of our data model to a greater extent is a natural direction for future work (see "Discussion" for details).

### Substantial growth in volume, connectivity, and hierarchical structure

The data model introduced in "Dynamic network model of legislation" enables us to track the development of our legislative corpora over time. As Table 1 shows, the absolute size of these corpora has grown substantially in the past two and a half decades, whether measured by the number of tokens (whitespace-delimited chunks of text that roughly correspond to words), the number of structural elements, or the number of cross-references contained therein. Judging merely by the number of tokens, in both jurisdictions, the law in 2018 is more than 1.5 times as large as the law in 1994. Given the fact that the legal systems of both countries were already fully developed twenty-five years ago, the sheer magnitude of this growth is striking.

**Table 1:** Federal legislation in the United States and Germany: descriptive statistics (1994 and 2018).

	United States			Germany		
	1994	2018	$\Delta$	1994	2018	$\Delta$
Tokens	14.0 M	21.2 M	$51~\%$	4.5 M	7.4 M	$64~\%$
Structures	452.4 K	828.1 K	$83~\%$	120.6 K	161.4 K	$34~\%$
References	58.0 K	88.6 K	$53~\%$	76.9 K	139.1 K	$81~\%$

**Figure 2:**
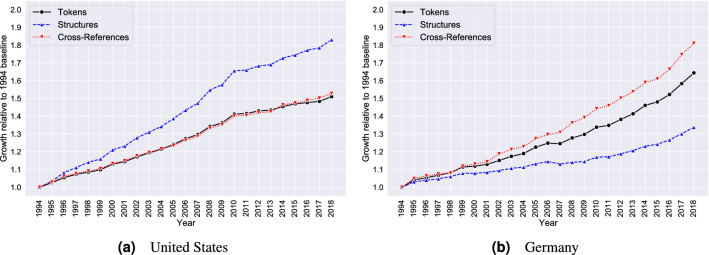
Federal legislation in the United States and Germany: growth statistics (1994–2018).

Inspecting the statistics in Table 1 along with the relative growth over time illustrated in Fig. 2 further reveals two distinct growth patterns: In the United States, the number of tokens and the number of cross-references grow at the same rate, which is considerably lower than the growth rate for the number of structural elements. In contrast, the German corpus exhibits its highest growth rate for the number of cross-references, and growth in the number of tokens is noticeably faster than growth in the number of structural elements. Thus, the volume increase in the federal statutory legislation of the United States is accompanied primarily by an increase in the number of entities, whereas the volume increase in the federal statutory legislation of Germany is accompanied primarily by an increase in the number of relationships in the legislative network. This suggests that cross-references and hierarchical elements function as substitutes when it comes to integrating new content into an existing legal corpus.

**Figure 3:**
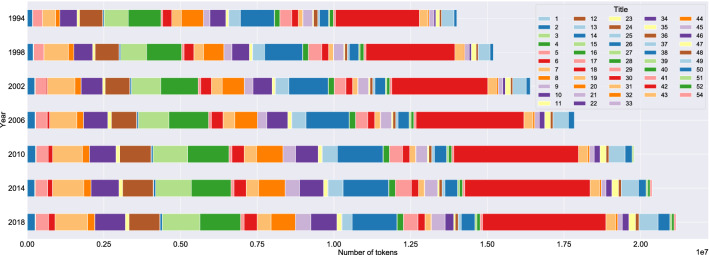
Federal legislation in the United States by Title (1994–2018), measured in tokens.

However, increasing the number of hierarchical elements or the number of cross-references also tends to correlate with a decrease in navigability as it may be indicative of content fragmentation: Anyone trying to understand a legal rule will more often be forced to combine information from multiple places in the law to obtain a complete picture of its content. This difficulty is only exacerbated by the dominant legal information systems, which often force users to click through hierarchies of legal elements and seldom allow them to display a custom selection of structural units in a single browser tab for joint appreciation. Therefore, our statistics for both countries support the intuition that their legislative apparatuses are growing also in complexity—although the complexity increase is driven by different design choices in both jurisdictions. While the difference in legislative drafting styles is of natural interest for comparative legal scholarship, the common growth trend we observe begs a broader question: What is its source?

This question has no meaningful answer within the current formal organisation of the legislative materials. In fact, the US Code as the primary organisational system for legislation in the United States has barely changed in the time period under study. The US Code comprised 50 Titles in 1994; since then, three Titles have been added (51, 52, and 54), two formerly empty Titles have been reassigned (6, 34), and two Titles have experienced small name changes (36, 47). Apart from that, US federal legislation has been codified in the same Titles since 1994, with the total number of Chapters existing across all Titles rising from 2000 to 2723 (for an average growth of 30 Chapters per year). Figure 3 localizes the growth over four-year intervals within the existing, content-based organisation of the US Code. Based on raw token counts (excluding notes and appendices), the biggest growth has occurred in Title 42 (The Public Health and Welfare), Title 7 (Agriculture), and Title 15 (Commerce and Trade). The relative growth in the number of tokens has been highest in Title 4 (Flag and Seal, Seat of Government, and the States), Title 46 (Shipping), and Title 7 (Agriculture).

**Figure 4:**
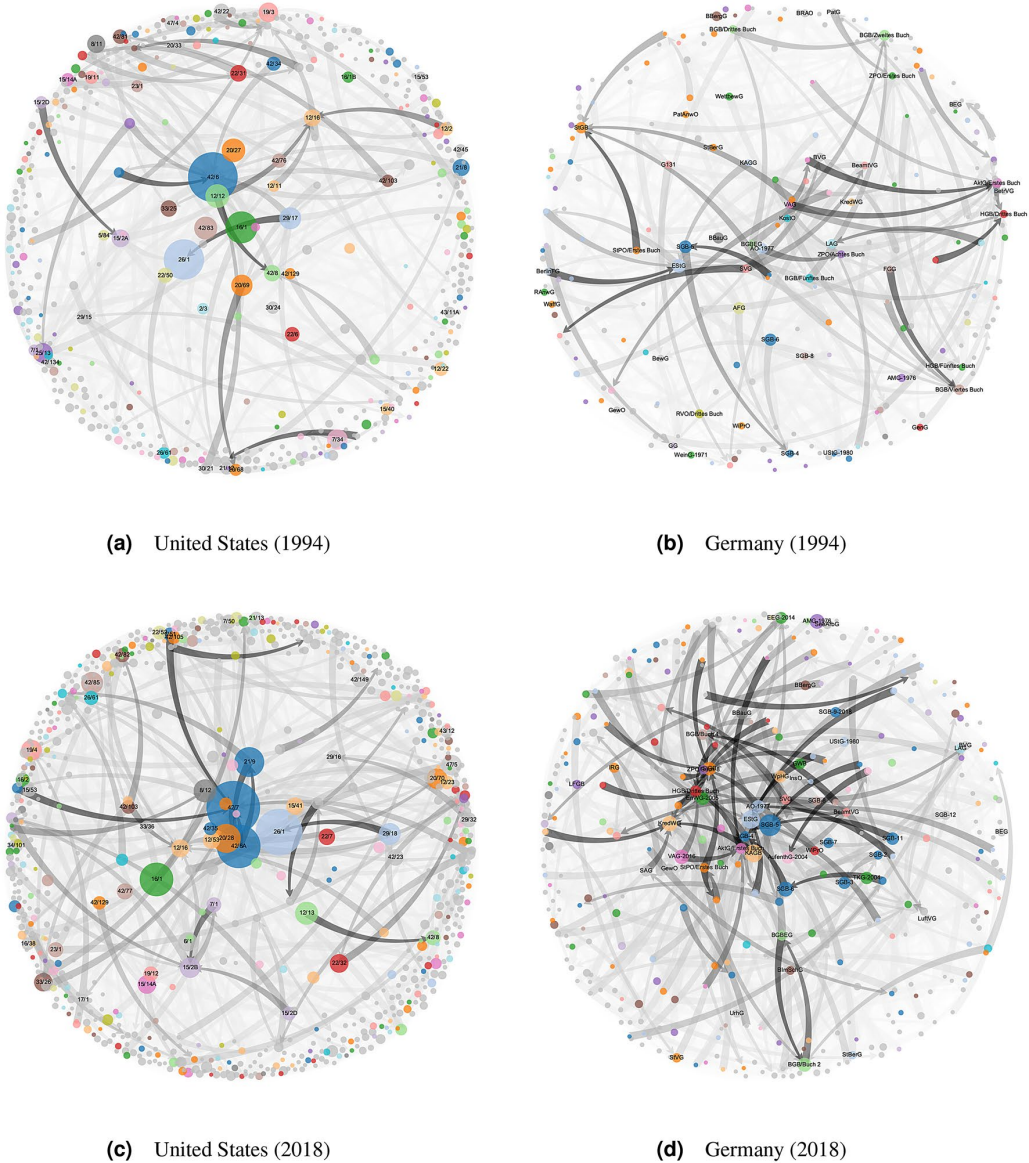
Federal legislation in the United States and Germany: quotient graphs by Title/Chapter (United States) and Law Name/Book (Germany) (1994 and 2018), with arrows running between nodes indicating that text contained in one node cites text contained in another node. Node sizes indicate token counts (larger = more tokens), where only nodes with at least 5000 tokens (corresponding to roughly ten pages) are shown. For each nation separately, nodes share the same colour if they are placed in the same cluster family, and nodes not in one of the 20 largest cluster families are coloured in grey. Only the labels of the 50 largest nodes (measured in tokens) are drawn.

This gives an interesting first impression on the macro level, but the Title headings are so general, and the content placed in the individual Titles is so diverse (e.g., the current Title 42 contains provisions on Social Security [Chapter 7], Energy Policy [Chapter 134], and Aeronautics and Space Activities [Chapter 155]), that it tells us little about the triggers and the nature of the growth we observe. The situation further deteriorates if we want to compare the German developments with those in the United States: Germany does not codify its federal legislation in a single official collection but publishes only individual acts and classifies them into subject areas for navigation (details can be found in Sect. 1.2 of the SI). The number of consolidated acts with more than 500 characters (roughly a paragraph, effectively excluding laws with purely formal content) grew from around 1550 to over 1800 in the period from 1994 to 2005, then was intentionally shrunk to around 1550 until 2011, and has resumed slow growth since 2011, reaching around 1600 in 2018—so we do not even see a monotone growth pattern in this data. To uncover the sources of the growth of the law, and compare our findings between the United States and Germany, we thus need to reorganise the legislative materials of both nations.

### Clustering for comparative and dynamic analysis

A first, straightforward way to reorganise the US Code is to aggregate it not at the Title level but rather at the Chapter level. This is especially convenient because the number of Chapters in the US Code is comparable to the number of individual laws in Germany, which we only break up into smaller units if they contain several Books (a common feature of large German codifications such as the German Civil Code [BGB] and the German Commercial Code [HGB]). The node-link diagrams of the quotient graphs corresponding to this reorganisation for the United States and Germany in 1994 and 2018 are shown in Fig. 4. In these graphs, nodes share the same colour if they belong to the same *cluster family*. Broadly speaking, cluster families are sets of clusters (a cluster is a set of nodes), mostly from different snapshots, which contain many identical, similar, or related rules (cf. Definition 8 in "Comparing document networks over space and time")—and as such, they approximate legal topics. We identify cluster families using node and cluster alignments (cf. "Comparing document networks over space and time"). Cluster families will help us assess which legal topics are driving the growth we report in "Substantial growth in volume, connectivity, and hierarchical structure". The cluster family colouring scheme will be used in all remaining graphics; a full legend mapping colours to legal topics can be found in Sect. 5.1 of the SI. In Fig. 4, nodes of the same colour can generally be thought of as belonging together (i.e., *same colour* $\Leftrightarrow$ *(roughly) same legal topic*), and node colours can be compared across years but not across nations (e.g., the legal topic of red nodes in the graphs for the United States may differ from the legal topic of red nodes in the graphs for Germany).

**Figure 5:**
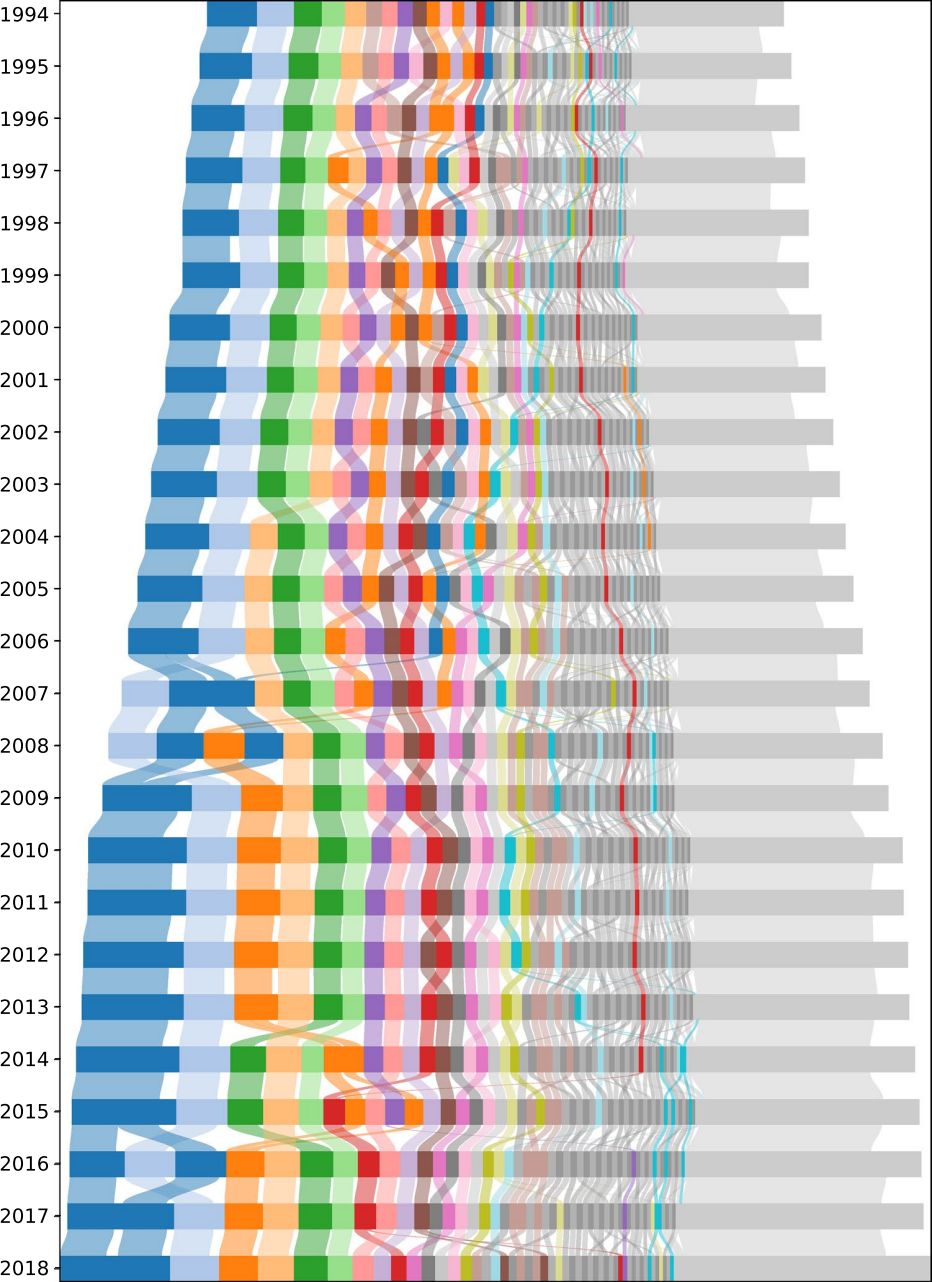
Federal legislation in the United States by cluster (1994–2018). Each block in each year represents a cluster. Clusters are ordered from left to right by decreasing size (measured in tokens) and coloured by the cluster family to which they belong, where clusters not in one of the 20 largest cluster families are coloured in alternating greys. Small clusters are summarised in one miscellaneous cluster, which is always the rightmost cluster and coloured in light grey. A full legend mapping colours to legal topics can be found in Sect. 5.1 of the SI.

The node-link diagrams in Fig. 4 allow us to identify interesting connections between individual parts of the law at the Chapter level— e.g., Book Three of the German Commercial Code (HGB/Drittes Buch), which regulates books of accounts, is much more central as a reference target in 2018 than it was in 1994, and the central role of Title 42, Chapter 6 of the US Code in 1994 (The Children’s Bureau) has been taken by Title 42, Chapter 6A (Public Health Service) and Chapter 7 (Social Security) in 2018. But since there are well over 1000 nodes in both jurisdictions, the quotient graphs are difficult to analyse in their entirety, related content remains scattered over different nodes, and the changes between snapshots are difficult to trace. Hence, the main conclusion from Fig. 4 is that the quotient graphs by Title/Chapter (United States) and Law Name/Book (Germany) alone are unsuited to unveil the temporal dynamics of legislative corpora in full detail. To coherently group related content and investigate change over time, we thus need a more sophisticated reorganisation method. Therefore, we cluster our annual Chapter quotient graphs for each country based on their cross-references.

**Figure 6:**
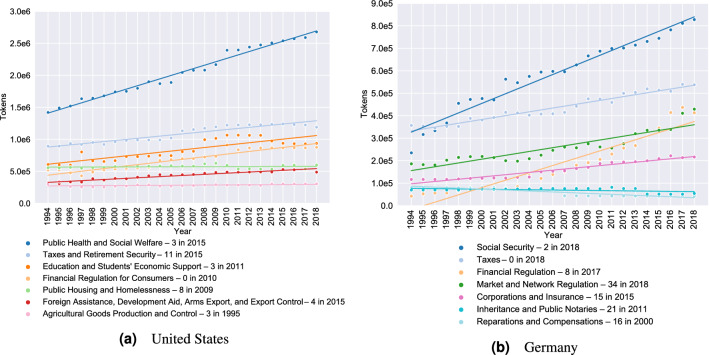
Federal legislation in the United States and Germany: growth statistics by cluster family for selected cluster families (1994–2018). The legends are sorted by the *y*-values of the regression lines in 2018. The colours are comparable across countries, i.e., *same colour* $\Leftrightarrow$ *(roughly) same topic*.

In (non-overlapping) graph clustering, the goal is to divide the elements of the graph (typically the nodes, here: Chapters in the United States and Books or individual laws in Germany) into groups such that elements in the same group are relatively densely connected, whereas elements in different groups are relatively sparsely connected. We use the *Infomap* algorithm based on the *map equation*^33,34^ to find our clusters for three reasons. First, in using random walks (i.e, sequences of random steps using the edges of the graph) as a basic ingredient, *Infomap* mimics how lawyers navigate legal texts. The legal navigation process is similar to how scholars navigate papers or web surfers navigate the World Wide Web (WWW), with the additional quirk that sequence edges play a large role in steering the search (think of reading the next paper in the special issue of a journal or clicking through a series of blog posts). Second, by leveraging the connection between finding clusters in a graph and minimizing the description length of a random walk on the graph, *Infomap* has a solid information-theoretic foundation. And third, the algorithm scales to large graphs.

When running *Infomap*, we use the default configuration, with one exception: We pass 100 as a parameter for the preferred number of clusters, which roughly corresponds to the number of top-level categories with which legal databases structure their content. This parameter choice allows us to determine the legal topics driving the growth we observe at a sufficiently high granularity while maintaining an overview of the entire corpus, and it protects against sudden jumps in the cluster granularity between years due to small differences in the description lengths of competing solutions, which are more likely to occur when no preferred cluster size is given. As detailed in the sensitivity analysis included in Sect. 4.1 of the SI, the precise number of input clusters has little impact on the overall results, as long as the numbers of clusters are comparable across years (e.g., tracing changes between a clustering in which most of the text is contained in 5 clusters and a clustering with 50 clusters is an invidious task). To increase the stability of our results, we obtain our final clustering for each country and year as the consensus clustering of 1000 *Infomap* runs, where the consensus clusters are the connected components of a graph whose nodes are the quotient graph nodes, and whose edges indicate which nodes co-occurred in the same cluster in $95~\%$ of all runs. As shown in Sect. 4.2 of the SI, there is little variance both across those runs and across multiple consensus clusterings using 1000 runs to find the consensus, indicating that our results are robust against the randomness inherent in the *Infomap* algorithm.

Based on our consensus clusterings, we can compute alignments between the clusters we find in subsequent snapshots for each of our countries. These *cluster* alignments allow us to track the temporal evolution of individual clusters. They are based on *node* alignments of a fine-grained variant of sequence graphs, which leverage that most rules do not change most of the time—i.e., we can match many *seqitems* between adjacent snapshots based on their (nearly) identical texts or (nearly) identical keys. For details on our node alignment heuristic and the cluster alignment procedure that builds upon it, see "Comparing document networks over space and time".

The fine-grained year-to-year cluster alignment facilitates a meso-level analysis of the growth reported in "Substantial growth in volume, connectivity, and hierarchical structure". Figure 5 provides a comprehensive overview of the aligned clusters for the entire United States corpus (an analogue figure for the German corpus can be found in Sect. 3.3 of the SI): The corpus in a certain year is modelled by a horizontal bar, which is composed of blocks representing clusters with width proportional to the number of tokens they contain. The year-to-year movement of tokens between clusters—i.e., the volume of text associated with one cluster in one year and another cluster in the next year, identified using the alignment between the items below the *seqitem* level (*subseqitems*) of the clusters—is indicated by splines connecting the blocks of adjacent years, where we only plot token movements corresponding to at least $15~\%$ of the tokens from both the ingoing and the outgoing cluster to filter out noise and isolate largely self-contained strands of the law as cluster families (see also "Comparing document networks over space and time"). The width of the plotted splines is again proportional to the number of tokens moving. Within each horizontal bar, the blocks representing the clusters are sorted in descending order by their size, i.e., the clusters with the largest numbers of tokens are always pushed to the left. To reduce visual clutter, we summarize very small clusters in one *miscellaneous* cluster. This cluster is always the rightmost cluster, depicted in light grey; more information on its contents can be found in Sect. 5.2 of the SI. The blocks and splines belonging to the 20 largest cluster families are uniquely coloured, whereas smaller cluster families are alternately coloured in alternating greys. The absolute growth of the United States corpus is reflected in the increasing width of the bars over time, whereas changes in cluster compositions and relative cluster sizes are visible as diagonal year-to-year movements.

Inspecting the numbers behind Fig. 5, we find that our clusters grow linearly with respect to their size, i.e., bigger clusters gain more tokens than smaller clusters, but that the growth rates differ depending on the legal topic represented by the cluster. To understand which legal topics are driving the overall growth, we determine the growth rate of our cluster families via an ordinary least squares (OLS) regression. We select the 20 largest cluster families for both countries, where the size of a cluster family is the size of its largest cluster (measured in tokens), called its *leading cluster*. For each of these cluster families, we inspect its content composition, and label it with the dominant legal topic. More information on our labelling process, including a list of all labels, can be found in Sect. 5.1 of the SI. Together, the labelled cluster families account for roughly $50~\%$ of the total growth in the United States and roughly $80~\%$ of the total growth in Germany. Figure 6 displays a selection of the most and least growing cluster families in the United States and Germany, while detailed results can be found in Sect. 3.4 of the SI. The colouring scheme for the United States is identical to that used in Fig. 5, while the colours for Germany are chosen to match those for the United States for similar topics and avoid colour clashes otherwise.

Notably, in both jurisdictions, growth rates are highest for the cluster families concerning social welfare and financial regulation, and cluster families dealing with taxes, environmental protection, and immigration also display strong growth in both countries. In addition to these similarities, we also find some differences in the growth patterns of both countries. As one might expect, the United States has cluster families concerned with Native Americans (shrinking) and student loans (growing), while no analogous families exist in Germany. Likewise, Germany has a cluster family concerned with war restitution (shrinking) that has no counterpart in the United States. The unmatched growth of the criminal law and corporate and insurance law cluster families in Germany, which may be counterintuitive at first sight, is probably a result of differences in legislative competencies (criminal law and corporate law including insurance are largely state law in the United States while they are federal law in Germany). In addition, insurance regulatory law on the federal level in the United States is primarily enforced through federal regulations, which are not part of our dataset as they are kept in a separate collection (the Code of Federal Regulations). That the United States has a growing cluster family concerned, inter alia, with foreign assistance and export control will not surprise those working in international development or international politics, and the fast-growing cluster dealing with renewable energy, power grid regulation, and related administrative procedures in Germany will not surprise those following the nation’s political discourse (although in both cases, the unexpectedness could be impacted by hindsight bias). Overall, the differences we observe seem to be in line with differences in the prominence of certain policy debates in both countries, reflecting social, political, and cultural divergences. As such, they invite in-depth analysis by subject matter experts.

Finally, the year-to-year cluster alignment underlying Fig. 5 allows us to observe different types of growth. For example, some clusters or cluster families witness *intrinsic growth*, i.e., growth by addition of tokens without large gains of tokens from other areas; the cluster family containing veteran’s benefits is a case in point, as is a cluster family on small business support and civil and military public procurement. Such cluster families, which have been rather self-contained in the past 25 years, address issues of sustained or increasing societal importance. Other clusters or cluster families, however, witness *extrinsic growth*, i.e., growth by gaining tokens from clusters in other families. One example is a United States cluster concerned with the environmental protection of national parks and rivers, which grew substantially when rules about national forests as well as prospecting permits and leases joined it from clusters concerned with forestry and mining, indicating a shift in perspective from land use as resource exploitation to land use as resource conservation. To capture such differences in change processes, an elaborate cluster change event taxonomy is needed. Such a taxonomy could build on the work by Palla et al.^4^, and developing it provides an interesting opportunity for further research.

## Discussion

This paper investigates the growth of federal legislation in two industrial countries over a period of 25 years. As such, it is limited in *geographic* scope (United States and Germany), *temporal* scope (1994–2018), and *institutional* scope (legislative bodies on the federal level). This makes it hard to assess to which extent the growth we observe is *particular* to our data or rather *universal*. The trend we identify applies to the recent history of federal legislation in at least two countries, the United States and Germany, and our findings in one country provide context for our findings in the other. Thus, we can establish that the growth we find is not a singular phenomenon, but we can only guess how it relates to the trends we might find in the legal document networks of other countries, time frames, or institutions.

The document networks that are most closely related to legislative networks are networks of regulations (produced by executive agencies) and networks of judicial decisions (produced by courts)—and for all of them, growth statistics that are directly comparable to ours are lacking. Some growth statistics are known for patent citation networks^35–38^, where, e.g., the number of patents granted annually by the United States Patent and Trademark Office has roughly tripled in the 25 years from 1994 to 2018^38^. Since the generating processes of patent citation networks are very different from those of legislative networks (patent applicants need to cite prior art in their filings, patent examiners can add further citations, and too much prior art might risk patentability) and the units of analysis are not the same (structural elements in legislative networks vs. individual patents in patent citation networks), however, this result has little bearing on our findings. For similar reasons, comparing our findings with results on non-legal document networks, such as the World Wide Web or scholarly networks, is potentially misleading. To put our findings into perspective, extending the scope of our data to other legal document networks is therefore an important direction for future work. For example, investigations in the following directions are supported by our legal network data model:

1. Analysing legislative activity on levels above and below the federal level and comparing the results with our findings will advance the search for invariants that characterize the development of legislative systems. It can also help us understand the division of labour within the legislative pyramid (e.g., the federal, the state, and the local level). Does state law grow even faster than federal law? If so, are the growth mechanisms similar or different? How do the answers to these questions depend on the allocation of legislative competencies?
2. Integrating documents from the executive and judicial branches of government with our datasets could help us explore how different parts of the legal system interact. How does the evolution of a legislative network compare to that of a network of administrative regulations, a network of executive orders, or a network of judicial decisions? In what areas of law is the development driven by the executive or the judiciary, rather than the legislative? What does this tell us about the distribution of power between the different branches of government?
3. Combining our legislative network data with data collected in other fields of quantitative social science might improve our understanding of the interaction between legal rules and other rule sets that impact the behaviour of individuals and societies. When, where, and how do legislative changes impact how people behave on the ground? When, where, and how do changes in how people behave prompt legislative changes? In other words: What *causal* relationships can we establish between legal change and societal change? These questions are inherently multidisciplinary, and to separate causes from confounders, legal network data would need to be combined with data reflecting public sentiment (e.g., social media data or public news data) and data reflecting individual or collective choices (e.g., financial network data, company reports, or economic panel data on households, firms, and non-governmental organisations). Similarly, a multi-pronged strategy could be pursued to investigate the relationships between legal change and *technological* change. Here, combining legal network data with patent citation network, patent litigation, and R&D investment data appears to be particularly promising.

Methodologically, our approach emphasizes the structural features of legislative texts. In particular, for the results we report in this work, the content of the legal texts has been only of indirect interest, e.g., as reflected in raw token counts or in reference structures that characterize legal topics. As demonstrated in "Clustering for comparative and dynamic analysis", however, qualitative analyses of the legal rules contained in our document networks can yield further insights, and this opens opportunities for normative legal research in areas such as comparative law and legal theory^39–41^. In these legal disciplines, the United States and Germany are usually classified as following different legal traditions, also referred to as *legal families*, and the categorization, though commonly accepted, has not been corroborated by empirical studies^42–45^.

Last but not least, the findings reported in this paper are based on a set of choices for methods and parameters. For example, we examine growth by analysing year-to-year net gain of tokens, as this difference can be determined reliably. The amount of legislative activity, however, is likely much higher (e.g., deletions and additions cancel out from the net gain perspective), and developing tools that allow for a fine-grained accounting of legislative changes constitutes an interesting research direction. While we explored our model space extensively (as detailed in Sect. 4 of the SI), the parametrisation of the clustering required numerous decisions based on our experience and familiarity with the subject matter. Other parametrisations are possible, and they might be needed in other analytical settings. In particular, future work could examine selected parts of our data in greater detail, zoom in on a particular legal topic, and therefore choose very different parameters to operate at a higher level of resolution.

## Methods

### Modelling legislative document collections

To formalize the intuition that is given in "Dynamic network model of legislation" and illustrated in Fig. 1, we use the following definitions. Let *D* be a document understood by the Document Object Model (DOM) standard, with elements ${\mathcal {E}}_D$ of types $\{$document, item, seqitem, subseqitem, text$\}$ and root $r_D$ of type document. We interpret *D* as a directed rooted tree $T_D$ in the graph theoretical sense, where the nodes of $T_D$ are the elements of *D* that are *not* of type text, and an arc between two nodes indicates that the source contains the target—i.e., $T_D$ contains all structural elements of *D* with their containment relations. With each node in $T_D$, we associate a unique identifier and three attributes: The *type* of a node is its type in *D*, the *level* of a node is its distance *d* from the root, with $d(r_D,r_D) = 0$, and the *text* of a node is the text of all its children (which can be used to derive additional statistics as necessary). Nodes of type seqitem (short for *sequence item*) typically have *cite keys*, i.e., sequentially ordered unique identifiers by which they are commonly referenced. All nodes may also have *headings* (representing the headings in the original document), and documents may have abbreviations by which they are commonly referenced. The custom XSD expressing this document model can be found in Sect. 2.4 of the SI.

Now let ${\mathcal {D}}^i_t$ be collection *i* of documents at time *t* with their tree representations ${\mathcal {T}}^i_t$. We define the following graphs for ${\mathcal {D}}^i_t$:

#### Definition 1

*(Hierarchy Graph* $H^i_t$*)* The *hierarchy graph* of collection ${\mathcal {D}}^i_t$, denoted $H^i_t$, is a *directed graph*

$$\begin{aligned} H^i_t = (V^i_{t,H}, E^i_{t,H})~, \end{aligned}$$

where

$$\begin{aligned} V^i_{t,H} = \underset{T\in {\mathcal {T}}^i_t}{\bigcup } V(T) \cup \{~{\bar{r}}_i~\} \end{aligned}$$

with a structural element ${\bar{r}}_i$ on level $-1$ representing the identity of the collection, and

$$\begin{aligned} E^i_{t,H} = \underset{T\in {\mathcal {T}}^i_t}{\bigcup } E(T) \cup \{~({\bar{r}}_i,r_D)\mid ~D\in {\mathcal {D}}^i_t~\}~. \end{aligned}$$

That is, the hierarchy graph is the union of all document trees’ structural elements equipped with their containment relation, joined by a meta root node identifying the collection.

#### Definition 2

*(Reference Graph* $R^i_t$*)* The *reference graph* of collection ${\mathcal {D}}^i_t$, denoted $R^i_t$, is a *directed multigraph*

$$\begin{aligned} R^i_t = (V^i_{t,H}, E^i_{t,R})~, \end{aligned}$$

where

$$\begin{aligned} E^i_{t,R} = E^i_{t,H}\cup C^i_{t}~, \end{aligned}$$

with $C^i_{t}$ a multiset given by

$$\begin{aligned} C^i_{t} =\{~(v,w)^m \mid ~\text {text of }v~\text {makes m references}\\\text {to}~w~\text {in }{\mathcal {D}}^i_t~\wedge ~type(v)=type(w)=\texttt {seqitem}~\}~. \end{aligned}$$

That is, the reference graph is the hierarchy graph, augmented by reference relations between its nodes.

#### Definition 3

*(Sequence Graph* $S^i_t(\rho , w, \alpha )$*)* The *sequence graph* of collection ${\mathcal {D}}^i_t$ with parameters $\rho$, *w*, and $\alpha$, denoted $S^i_t(\rho , w, \alpha )$, is a *directed multigraph*

$$\begin{aligned} S^i_t(\rho , w, \alpha ) = (V^i_{t,S}(\rho ), E^i_{t,S}(\rho , w, \alpha ))~. \end{aligned}$$

Here, $V^i_{t,S}(\rho )$ initially contains all nodes of type seqitem, and nodes that are neighbours in the sequence are merged if and only if they meet the *merge condition* $\rho$. $E^i_{t,S}(\rho , w, \alpha )$ contains the arcs of $R^i_t$, projected onto the node set of $S^i_t$, with containment relations now represented as a pair of sequence arcs between nodes with adjacent *cite keys*. The sequence arcs in $E^i_{t,S}(\rho , w, \alpha )$ are weighted according to a weight function *w* (specifying the weight decay of sequence arcs as a function of the distance between the source node and the target node in the undirected graph underlying $H^i_t$), and the reference arcs are weighted according to a weight ratio $\alpha$ (specifying the weight of reference arcs in relation to sequence arcs of maximum weight).

As mentioned in "Dynamic network model of legislation", the sequence graph representation of a legislative document collection is inspired by how practitioners work with legislative texts. Furthermore, the parameters of the sequence graph allow us to incorporate knowledge about legal users into our model (e.g., by weighting reference arcs less heavily than the highest-weight sequence arcs, we can express the intuition that looking up a reference is less likely than simply reading on). To compute the node alignments mentioned in "Clustering for comparative and dynamic analysis", we use a more granular variant of the sequence graph:

#### Definition 4

*(Subsequence Graph* ${\bar{S}}^i_t(\rho , w, \alpha )$*]* The *subsequence graph* of collection ${\mathcal {D}}^i_t$ with parameters $\rho$, *w*, and $\alpha$, denoted ${\bar{S}}^i_t(\rho , w, \alpha )$, is defined as the sequence graph $S^i_t(\rho , w, \alpha )$, with *seqitems* being replaced by *subseqitems* (i.e., structural elements one level below the seqitem level) if they exist.

Finally, we use a multigraph version of the standard graph theoretical notion of a quotient graph (see also "Comparing document networks over space and time"):

#### Definition 5

*(Quotient Graph* *Q*(*G*, *R*)*)* Given a graph *G* and an equivalence relation *R* on its node set *V* (i.e., a reflexive, symmetric, and transitive binary relation), a quotient graph is the graph *Q*(*G*, *R*) with

$$\begin{aligned} V_{Q(G,R)} = V / R = \{~[u]_R \mid u\in V~\} \end{aligned}$$

and

$$\begin{aligned} E_{Q(G,R)} = \{~([u]_R, [v]_R)^m \mid ~|\{~(x,y) \in E_G \mid x\in [u]_R \wedge y\in [v]_R~\}| = m > 0~\}~, \end{aligned}$$

where $[u]_R := \{~x\in V\mid (u,x)\in R~\}$ and $[v]_R := \{~y\in V\mid (v,y)\in R~\}$ are equivalence classes of *V* under *R*.

As shown in "Clustering for comparative and dynamic analysis" for aggregating legal texts at the Chapter level, the equivalence relations of our quotient graphs are generally given by the attributes associated with the structural elements contained in our reference graphs. Another example of quotient graphs, based on the cluster identifiers produced by our graph clustering as node attributes, can be found in Sect. 3.2 of the SI.

### Assessing legislative growth

To assess legislative growth in "Clustering for comparative and dynamic analysis", we track three statistics for the United States and Germany from 1994 to 2018: the number of tokens, the number of hierarchical structures, and the number of references contained in the federal statutory legislation of both countries. For the token counts, we concatenate the text of all statutory materials for one country and year, ignoring the extensive appendices to some Titles or laws, and split on whitespace characters. The hierarchical structure counts reflect the number of nodes in our hierarchy graphs, and the reference counts reflect the number of edges in our reference graphs. Details on our data preprocessing steps can be found in Sect. 2 of the SI.

### Comparing document networks over space and time

#### Clustering document networks

To enable our comparative and dynamic analysis in "Clustering for comparative and dynamic analysis", we cluster each annual snapshot of the legislative network separately for both countries. As mentioned in "Clustering for comparative and dynamic analysis", amongst the plethora of graph clustering methods, we choose the *Infomap* algorithm due to its information-theoretical underpinnings, scalability, and interpretability as a legal (re-)search process. Details on this algorithm can be found in the original papers^33,34^.

As the input data to *Infomap*, we use the sequence graph representation of an annual snapshot with a merge condition $\rho$ that collapses into one node all rules from the same Chapter (or Title, if the Title has no Chapters) in the United States, and all rules from the same Book (or law, if the law has no Books) in Germany. This consolidation step densifies the adjacency matrix of the sequence graph and reduces the noise in our data. As almost all remaining nodes lie at distance 2 from one another in the hierarchy graph, and very few sequence edges would remain, we base the clustering solely on references. Legislative network analyses using a different $\rho$ would also require the choice of a weight decay function *w* and a *sequence edge*-to-*reference edge* weight ratio $\alpha$. For *Infomap* itself, we use the default configuration with a preferred cluster number of 100 as an additional input parameter. As discussed in "Clustering for comparative and dynamic analysis", this parameter choice reflects the level of analytical resolution we seek to operate at, and it approximates the number of high-level topics legal database providers utilise to organise their content. The sensitivity analysis regarding our input parameter can be found in Sect. 4.1 of the SI.

As *Infomap* has a stochastic element, we use *consensus clustering*^46^ to increase the robustness of our results as follows: For each snapshot *t* in each country *i*, we produce 1000 clusterings with different seeds. From the results of these clusterings, we produce a *consensus graph* whose nodes are the nodes of the sequence graph, and with an edge connecting two nodes if these nodes are in the same cluster in at least $950 = 95~\%$ of our *Infomap* runs. For each year and country, the connected components of the consensus graph then constitute our final clusters, which represent a careful reorganisation of the law enabling comparative and dynamic analysis. This leads to more than 100 final clusters because the initial clusters are typically split into a stable core and several smaller satellites, each of which becomes an additional separate cluster.

#### Tracing temporal dynamics

To trace legislative change over time, we need to align the textual contents of our yearly snapshots within each jurisdiction. Computing the optimal node alignment between two graphs is generally a hard problem, and methods based on tree edit distance do not scale to legislative trees. However, we can use sequence graphs with the highest possible granularity (using a merge condition $\rho$ that condenses nothing) along with the text associated with individual nodes, and exploit the fact that most rules do not change most of the time to construct a practical heuristic that greedily computes a partial node alignment $\phi ^i_t$ across two snapshots $S^i_t$ and $S^i_{t+1}$ from corpus *i*. Our heuristic operates in at most four sequential passes through these snapshots:

1. First pass: If *v* is a node in $S^i_t$ and we find exactly one node *w* in $S^i_{t+1}$ with identical text *and* the text is at least 50 characters long, set $\phi ^i_t(v) = w$.
2. Second pass: If *v* is an unmatched node in $S^i_t$ and we find an unmatched node *w* in $S^i_{t+1}$ with identical key *and* identical text, set $\phi ^i_t(v) = w$.
3. Third pass: If *v* is an unmatched node in $S^i_t$ and we find exactly one unmatched node *w* in $S^i_{t+1}$ such that (i) the text of *v* contains the text of *w* (or the text of *w* contains the text of *v*) and (ii) the text remaining unmatched in *v* (*w*) is shorter than the matched part, set $\phi ^i_t(v) = w$.
4. Fourth pass: If *v* is an unmatched node in $S^i_t$ and we find a matched node $v'$ in $S^i_t$ in the five-hop neighbourhood of *v*, search the five-hop neighbourhood of $\phi ^i_t(v')$ for the unmatched node *w* (if any) with the largest Jaro-Winkler string similarity^47^ to *v*; if that similarity is above 0.9, set $\phi ^i_t(v) = w$. Repeat recursively with all newly matched nodes until no further matches are found.

With this procedure, we manage to map between $94~\%$ and $100~\%$ of the *subseqitems* between adjacent snapshots in both the United States and Germany, i.e., our partial node alignments are almost complete, and the unmatched subseqitems are indicators of larger changes in the code (rather than errors). Based on partial *node* alignments $\phi ^i_t$ for all relevant *t*, we compute a partial *cluster* alignment across snapshots, which we call the *cluster graph* $C^i$:

##### Definition 6

*(Cluster Graph* $C^i$*)* Let $C^i_t$ be the consensus clustering obtained for collection *i* at time *t*. The *cluster graph* of collection *i* across times *T*, denoted $C^i$, is a weighted digraph

$$\begin{aligned} C^i = (V^i_C,E^i_C)~, \end{aligned}$$

where

$$\begin{aligned} V^i_C = \underset{t \in T}{\bigcup } \{~c \in C^i_t~\} \end{aligned}$$

and

$$\begin{aligned} E^i_C = \{~(c,c',w) \mid c\in C^i_t~\wedge ~c'\in C^i_{t+1}~\wedge ~ \Delta (c,c') = w~\} \end{aligned}$$

with

$$\begin{aligned} \Delta (c,c') = \sum _{v~\in ~c~\setminus ~\{~v~\mid ~\phi ^i_t(v)~\notin ~c'~\}}|\phi ^i_t(v)|~, \end{aligned}$$

where |*v*| denotes the number of tokens in a node *v* of the sequence graph $S^i_t$ used as input to the clustering at time *t*.

That is, the cluster graph $C^i$ contains the clusters resulting from the clusterings of all snapshots as nodes, and its weighted edges $(c,c',w)$ indicate how many tokens from a cluster $c'\in C^i_{t+1}$ stem from cluster $c\in C^i_t$.

The cluster graph allows us to identify substantial additions, deletions, and movements of tokens in the United States and Germany over our entire period of study, revealing dynamics at the level of *individual clusters*. To trace dynamics at the level of *legal topics*, we define *cluster families* based on the *family graphs* of our collections:

##### Definition 7

*(Family Graph* $F^i(\gamma )$*)* Let $C^i$ be the cluster graph of collection *i* across times *T*. The *family graph* of collection *i* across times *T*, denoted $F^i$, is a weighted digraph

$$\begin{aligned} F^i(\gamma ) = (V^i_C,E^i_F(\gamma ))~, \end{aligned}$$

where

$$\begin{aligned} E^i_F(\gamma ) = \{~(c,c',w)~\mid ~(c,c',w) \in E^i_C~\wedge ~\chi (c,c',w) \ge \gamma ~\} \end{aligned}$$

with

$$\begin{aligned} \chi (c,c',w)=\min ~\Big \{~\frac{w}{|c|}, \frac{w}{|c'|}~\Big \}~, \end{aligned}$$

where |*c*| denotes the number of tokens in cluster *c*.

In words, the family graph $F^i(\gamma )$ contains the same nodes as the cluster graph $C^i$ but only those edges from $(c,c',w)~\in ~E^i_C$ that account for at least a $\gamma$ fraction of the tokens in both *c* and $c'$. We set $\gamma = 0.15$ to filter out noise and isolate parts of the cluster graph that are largely self-contained, but this threshold can be replaced by any other number between 0 and 1 for other analyses.

To trace the evolution of legal topics over time, based on the family graph, we define:

##### Definition 8

*(Cluster Family* $V^i_{F,j}$*)* Let $F^i(\gamma )$ be the family graph for collection *i* across times *T* consisting of cluster families as connected components. A *cluster family* $V^i_{F,j}$ is the node set of $F^i(\gamma )$’s *j*^th^ largest connected component (measured in tokens).

In addition to the overall size of a cluster family (given by the size of its leading cluster), our analysis also uses a temporal notion of cluster family size:

##### Definition 9

*(Cluster Family Size at Time* *t* $|V^i_{F,j,t}|$*)* Let $V^i_{F,j}$ be a cluster family *j* in collection *i*, and let $C^i_t$ be the consensus clustering obtained for time *t*. The size of cluster family *j* at time *t* is defined as

$$\begin{aligned} |V^i_{F,j,t}| = \sum _{c~\in ~(V^i_{F,j}~\cap ~C^i_t)}|c|~, \end{aligned}$$

where |*c*| denotes the number of tokens in a node *c*.

With our parametrisation, cluster families are sets of Chapters, Books, or laws that are closely related by cross-references or (almost) textual identity over time. As such, they approximately correspond to *legal topics*. Further information on how we label these topics can be found in Sect. 5.1 of the SI.

## Supplementary information

Supplementary Information.

## Data Availability

For the United States, the raw input data used in this study is publicly available from the Annual Historical Archives published by the Office of the Law Revision Counsel of the U.S. House of Representatives, and is also available from the authors upon reasonable request. For Germany, the raw input data used in this study was obtained from *juris GmbH* but restrictions apply to the availability of this data, which was used under license for the current study, and so is not publicly available. For details, see Sect. 1.2 of the SI. The preprocessed data used in this study (for both the United States and Germany) is archived under the following DOI: 10.5281/zenodo.4070767.

## Code availability

The code used in this study is available on GitHub in the following repositories: Paper—https://github.com/QuantLaw/Complex-Societies-and-Growth; DOI of publication release: 10.5281/zenodo.4070769. Data preprocessing—https://github.com/QuantLaw/legal-data-preprocessing; DOI of publication release: 10.5281/zenodo.4070773. Clustering—https://github.com/QuantLaw/legal-data-clustering; DOI of publication release: 10.5281/zenodo.4070775.
