# Supplementary material for: Complex societies and the growth of the law
Source: Sci Rep. 2020 Oct 30;10:18737. doi: 10.1038/s41598-020-73623-x (PMC7599339; doi:10.1038/s41598-020-73623-x)
Supplement: Supplementary file 1 — Supplementary Information. [file 41598_2020_73623_MOESM1_ESM.pdf]

Complex Societies and the Growth of the Law

Daniel Martin Katz[1,2,5,*], Corinna Coupette[3], Janis Beckedorf[4], and Dirk Hartung[2,5]

[1]Illinois Tech – Chicago Kent College of Law
[2]CodeX – The Stanford Center for Legal Informatics
[3]Max Planck Institute for Informatics
[4]Faculty of Law, Ruprecht-Karls-Universität Heidelberg
[5]Bucerius Law School
[*]dkatz3@kentlaw.iit.edu

SUPPLEMENTARY INFORMATION

1 Data sources

1.1 United States

For the United States, we use the United States Code (US Code) as our data source. The US Code is a compilation of the general and permanent laws of the United States on the federal level, excluding state legislation. The Office of the Law Revision Counsel of the U.S. House of Representatives (Office) updates the code continuously and publishes annual versions. When Congress passes new legislation, this legislation is initially published as a *Slip Law* in the *United States Statutes at Large*. If the new legislation is considered general and permanent law, the Office integrates the law into the US Code. Depending on the Titles of the Code that are modified, the work of the Office is approved by Congress, whereby the *Slip Law* is repealed and replaced by the US Code as the new primary source of the law. In other cases, the US Code is still presumed to be the correct consolidation of the law.

We base our work on the Annual Historical Archives published by the Office, which are available on its website: `https://uscode.house.gov/download/annualhistoricalarchives/annualhistoricalarchives.htm`.
The US Code is provided in (X)HTML format as documented at `https://uscode.house.gov/download/resources/USLM-User-Guide.pdf`. The format is flexible and offers a wide variety of styles to closely represent the printed code. The code is split into single files per year and Title. The Annual Historical Archives date back until 1994, and they are published with delay. As of 2020, therefore, 2018 is the latest available edition/supplement.

While surveying and validating the raw data, we observe and correct the following obvious errors:
– double-closing `<div>`-Tags in Title 40 in 2008, and in Title 42 in 1994 and 1995,
– inconsistent metadata in the appendix of Title 28 in 2017,
– a duplicate of Title 12 in 1998 that is included in files regarding Title 11 and 12, and
– inconsistencies regarding the tags `<statute>` and the comments `<!- section-head ->`.

1.2 Germany

In Germany, all laws are published in the Federal Law Gazette as amending laws, which often combine numerous introductions of new as well as changes to and repeals of existing laws. Individual laws are officially classified into one of nine substantive categories with currently 73 sub-categories containing over 400 subject areas in the “Fundstellennachweis A” [engl. Finding Aids A]. These are published on a yearly basis by the Federal Law Gazette upon instruction by the Ministry of Justice. However, unlike the US Code, there is no official data source that provides all compiled general and permanent laws at the federal level and their historical versions. Therefore, we cooperate with the leading German legal data

provider, *juris GmbH*, to obtain a dataset similar to the annual versions of the US Code. Although *juris* is a private company, the Federal Republic of Germany is its majority shareholder, and all branches of government rely heavily on *juris* to process legislative data. According to *juris*, the database contains every German federal law since spring 1990. Compared to the US Code, the data is not as structured. Instead of providing annual consolidated versions, *juris* provides a new version of a law for all changes that take effect on the same day. The data we obtained comes in separate files for each law and version.

We may not share the text content of the German dataset together with this paper. However, a website maintained by the German Federal Ministry of Justice and Consumer Protection in collaboration with *juris* (https://www.gesetze-im-internet.de) provides almost the entire federal legislation as XML files in the most recent version (i.e., without historical versions). A daily archive of the XML files provided on this website (starting in June 2019) is available at https://github.com/QuantLaw/gesetze-im-internet. This dataset allows a partial reproduction of the research with a similar dataset. We requested the full dataset from *juris GmbH*, which required a dedicated contractual framework and non-disclosure agreement to be signed.

1.3 Samples of Legal Texts

The following fragments illustrate the inherent structure of legal texts. Inclusion relationships are marked by indentation, labels of *seqitems* are typeset in SMALL CAPITALS, cross-references are underlined, and text content is set in *Italics*.

United States Code (2018 Main Edition dated January 14, 2019)

Title 12—Banks and Banking

Chapter 42—Low-Income Housing Preservation and Resident Homeownership

Subchapter I—Prepayment of Mortgages Insured under National Housing Act

§4101. GENERAL PREPAYMENT LIMITATION

(a) Prepayment and termination

An owner of eligible low-income housing may prepay, and a mortgagee may accept prepayment of, a mortgage on such housing only in accordance with a plan of action approved by the Secretary under this subchapter or in accordance with section 4114 of this title. An insurance contract with respect to eligible low-income housing may be terminated pursuant to section 1715t of this title only in accordance with a plan of action approved by the Secretary under this subchapter or in accordance with section 4114 of this title.

...

...

§4105. FEDERAL COST LIMITS AND LIMITATIONS ON PLANS OF ACTION

(a) Determination of relationship to Federal cost limits

(1) Initial determination

For each eligible low-income housing project appraised under section 4103(a) of this title, the Secretary shall determine whether the aggregate preservation rents for the project determined under paragraph (1) or (2) of section 4104(b) of this title exceed the amount determined by multiplying 120 percent of the fair market rental (established under section 1437f(c) of title 42) for the market area in which the housing is located by the number of dwelling units in the project (according to appropriate unit sizes).

German Civil Code (official translated version dated October 1, 2013)

Book 1—General Part

Division 1—Persons

Title 1—Natural persons, consumers, entrepreneurs

SECTION 1—BEGINNING OF LEGAL CAPACITY

The legal capacity of a human being begins on the completion of birth.

SECTION 2—BEGINNING OF MAJORITY

Majority begins at the age of eighteen.

...

Title 2—Legal persons

Subtitle 1—Associations

Chapter 1—General provisions

...

SECTION 40 —FLEXIBLE PROVISIONS

The provisions of section 26 (2) sentence 1, section 27 (1) and (3), sections 28 and 31a (1) sentence 2, as well as sections 32, 33 and 38, do not apply where otherwise provided by the articles of association. It is not possible to derogate from section 34 through the articles of association, even for the passing of resolutions by the board.

2 Data preprocessing

We convert the source data into a structured format that facilitates access for our purposes, removes unnecessary details (especially most of the style information for the text), and unifies the data format across both countries. For the purposes of our analysis, we focus on three properties that characterise the structure of legislative texts (illustrated in Section 1.3 above):

1. *Hierarchy:* They are nested, e.g., into Titles, Sections, Subsections, Paragraphs, and Subparagraphs.
2. *Reference:* They are interlinked, e.g., one Section can reference (the label of) another Section in its text.
3. *Sequence:* They are ordered, e.g., Sections have unique labels, and they appear in the text in ascending order of their labels.

To make these properties easily accessible in our data, we perform the following preprocessing steps for each dataset:

2.1 Clean the text

First, we remove all formatting, annotations, notes, and metadata from the text, with the exception of formatting and metadata that we need to extract the hierarchy in the next step. For the United States, this results in a more conservative definition of legal text than in [1], which explains the difference between the reported token counts. As the German data is not consistently formatted on a more detailed level than text paragraphs (meaning one level below § or Articles), we do not preserve formatting below the paragraph level for this dataset.

2.2 Extract the hierarchies

Using the remaining text, formatting, and metadata, we extract the hierarchy, i.e., we identify boundaries of elements (Chapters, Parts, Sections, etc.) that structure the code, determine their parents (Titles, Chapters, Parts, etc.) and, if present, their headings (including the alphanumeric ordering identifier), and retrieve their children or textual content. With this information, we generate an XML representation of the code in which the text and structural elements are nested inside their respective parents.

Our data contains explicit information regarding the boundaries and nesting of structural elements above the Section level in its metadata. We rely on this metadata down to the Paragraph level in Germany and down to the Section level for the United States. Below the Section level in the United States, we exploit the formatting to derive a nesting of the text. Extracting the hierarchy information from the metadata yields all information required to build our hierarchy graphs.

2.3 Extract the cross-references

Next, we extract all explicit cross-references in the statutory texts that match a common citation format. To simplify the process, we perform the extraction in three steps:

1. *Find*: We identify parts of the text that contain a potential reference to another text in the same statutory collection. We define country specific regular expressions (*regex*) patterns to find the referencing parts.

   Our pattern for the US Code is rather simple, as references are mostly formatted consistently and include no headings or names but only numbers (and potentially letters of alphanumeric enumerations). The pattern for Germany is more sophisticated. The start of a reference is easy to identify as references normally begin with "§" or "Art.". The part of the reference that follows may contain numbers (and letters of alphanumeric enumerations) as well as units (e.g., Satz [engl. sentence], Nummer [engl. number]). In the case of a reference to a text in a different law, the reference is followed by the name of the law. A list of the law names is generated from the source data, but it includes only laws valid at the time the analysed law takes effect. Furthermore, references to other national regulations, EU legislation, etc., are filtered out, so that only references within a law and to other laws that are part of the collection remain. Detailed documentation regarding the citation format in German laws can be found in the "Handbuch der Rechtsförmlichkeit" [engl. Handbook of Legal Formalism] of the Federal Ministry of Justice and Consumer Protection: `https://www.bmjv.de/DE/Themen/RechtssetzungBuerokratieabbau/HDR/HDR_node.html`. Since this guide is not strictly followed by the legislator, we used this guide along with the actual data to develop our extraction method.

2. *Parse*: We parse the referencing texts and derive citation keys (*cite keys*) that, for the US Code, consist of a Title and a Section of the referenced text. In the German case, the keys are composed of the abbreviation of the referenced law and the number of the cited § or Article. One reference identified in the first step may contain several such citation keys.

3. *Align*: We identify the target structural elements of the parsed references. To accomplish this, we generate citation keys for each Section, §, or Article that can be referenced by a specific version of a law. In the United States, a Section can be referenced if its structural element is part of the same annual version of the US Code. In Germany, the structural element must be part of a valid law when the analysed law takes effect.

2.4 Generate XML files

We store each preprocessed Title in the United States and each law in Germany in a separate XML file. The XML files comply with the following XSD specification:

```
<xs:schema
  attributeFormDefault="unqualified"
  elementFormDefault="qualified"
  xmlns:xs="http://www.w3.org/2001/XMLSchema"
>
  <xs:element
    name="document"
    type="documentType"
  />
  <xs:complexType name="documentType">
    <xs:choice
      maxOccurs="unbounded"
      minOccurs="0"
    >
      <xs:element
        type="itemType"
        name="item"
      />
      <xs:element
        type="seqitemType"
        name="seqitem"
      />
    </xs:choice>
    <xs:attribute
      type="xs:string"
      name="heading"
      use="required"
    />
    <xs:attribute
      type="xs:string"
      name="key"
      use="required"
    />
    <xs:attribute
      type="xs:unsignedInt"
      name="level"
      use="required"
    />
    <xs:attribute
      type="xs:string"
      name="abbr_1"
      use="optional"
    />
    <xs:attribute
      type="xs:string"
```

```
      name="abbr_2"
      use="optional"
    />
  </xs:complexType>
  <xs:complexType name="itemType">
    <xs:choice
      maxOccurs="unbounded"
      minOccurs="0"
    >
      <xs:element
        type="itemType"
        name="item"
      />
      <xs:element
        type="seqitemType"
        name="seqitem"
      />
    </xs:choice>
    <xs:attribute
      type="xs:string"
      name="heading"
      use="required"
    />
    <xs:attribute
      type="xs:string"
      name="key"
      use="required"
    />
    <xs:attribute
      type="xs:unsignedInt"
      name="level"
      use="required"
    />
  </xs:complexType>
  <xs:complexType name="seqitemType">
    <xs:sequence>
      <xs:element
        type="subseqitemType"
        name="subseqitem"
        maxOccurs="unbounded"
        minOccurs="0"
      />
      <xs:element
        type="textType"
        name="text"
        maxOccurs="unbounded"
        minOccurs="0"
      />
    </xs:sequence>
```

```
  <xs:attribute
    type="xs:string"
    name="citekey"
    use="optional"
  />
  <xs:attribute
    type="xs:string"
    name="heading"
    use="optional"
  />
  <xs:attribute
    type="xs:string"
    name="key"
    use="required"
  />
  <xs:attribute
    type="xs:unsignedInt"
    name="level"
    use="required"
  />
</xs:complexType>
<xs:complexType name="subseqitemType">
  <xs:sequence>
    <xs:element
      type="textType"
      name="text"
    />
  </xs:sequence>
  <xs:attribute
    type="xs:string"
    name="key"
    use="required"
  />
  <xs:attribute
    type="xs:byte"
    name="level"
    use="required"
  />
</xs:complexType>
<xs:complexType
  name="textType"
  mixed="true"
>
  <xs:sequence>
    <xs:element
      type="referenceType"
      name="reference"
      maxOccurs="unbounded"
      minOccurs="0"
```

```
      />
    </xs:sequence>
  </xs:complexType>
  <xs:complexType name="referenceType">
    <xs:sequence>
      <xs:element
        type="xs:string"
        name="main"
      />
      <xs:element
        type="xs:string"
        name="suffix"
      />
      <xs:element name="lawname">
        <xs:complexType>
          <xs:simpleContent>
            <xs:extension base="xs:string">
              <xs:attribute
                type="xs:string"
                name="type"
                use="optional"
              />
            </xs:extension>
          </xs:simpleContent>
        </xs:complexType>
      </xs:element>
    </xs:sequence>
    <xs:attribute
      type="xs:string"
      name="parsed"
      use="optional"
    />
    <xs:attribute
      type="xs:string"
      name="parsed_verbose"
      use="optional"
    />
    <xs:attribute
      type="xs:string"
      name="pattern"
      use="optional"
    />
  </xs:complexType>
  <xs:complexType name="lawnameType">
    <xs:simpleContent>
      <xs:extension base="xs:string">
        <xs:attribute
          type="xs:string"
          name="type"
```

```
            use="required"
          />
        </xs:extension>
      </xs:simpleContent>
    </xs:complexType>
</xs:schema>
```

2.5 Generate graphs

We use the XML files along with information regarding the annual version the file belongs to (in the United States) or the validity period of a specific version of a law (in Germany) to generate our hierarchy graphs and our reference graphs. We produce these graphs for each annual version of the US Code, and for snapshots of the German data taken at the first day of each year from 1994 to 2018. For the German data, we can generally produce hierarchy graphs and reference graphs for each day in the period under study; the chosen dates are designed to match the United States data. The sequence graphs and the quotient graphs are derived from the reference graphs as simple transformations. All our graphs are generated using NetworkX (https://networkx.github.io) and can be exported, e.g., as GraphML files (http://graphml.graphdrawing.org/specification.html).

3 Figure generation

3.1 Main paper Figure 3

Figure 3 from the main paper visualises the size of individual Titles of the US Code for every fourth year, starting from 1994. Title 53, which is missing in the legend, has been empty throughout the period under study. In the main paper, we illustrate the size of Titles based on tokens. In Figure 1, we show the size of Titles based on other measures, namely, structural elements, outgoing references to other Titles, ingoing references from other Titles, and internal references.

In all of the graphics, 12 colours rotate to mark the different Titles. Since colours are reused, the position of a bar must be taken into account when reading the legend. The Titles are plotted in a horizontally stacked bar chart, following their original order from left to right, i.e., starting with Title 1 on the very left.

3.2 Main paper Figure 4

Figure 4 from the main paper shows graphs representing the Chapters of the US Code or German laws as nodes. If a German law contains Bücher (engl. Books) at the highest hierarchy level, we split the law into its Books and use the Books as nodes. Arrows between nodes indicate that the text of one node cites the text of another. The opacity of an arrow indicates the number of references that are summarised in this arrow. These nodes and weighted edges visualise the major part of the data we run the *Infomap* clustering algorithm on. However, here, we hide nodes containing less than 5000 tokens (which are included for the clustering).

In the figure, we colour nodes by their cluster family, and colours are comparable across years but not across countries. We encode the number of tokens contained in a node by its area, which is comparable between the four graphs in Figure 4 from the main paper. The opacity of edges is individually scaled for each graph to cover the full range of opacity. The minimal and maximal values can be found in Table 1.

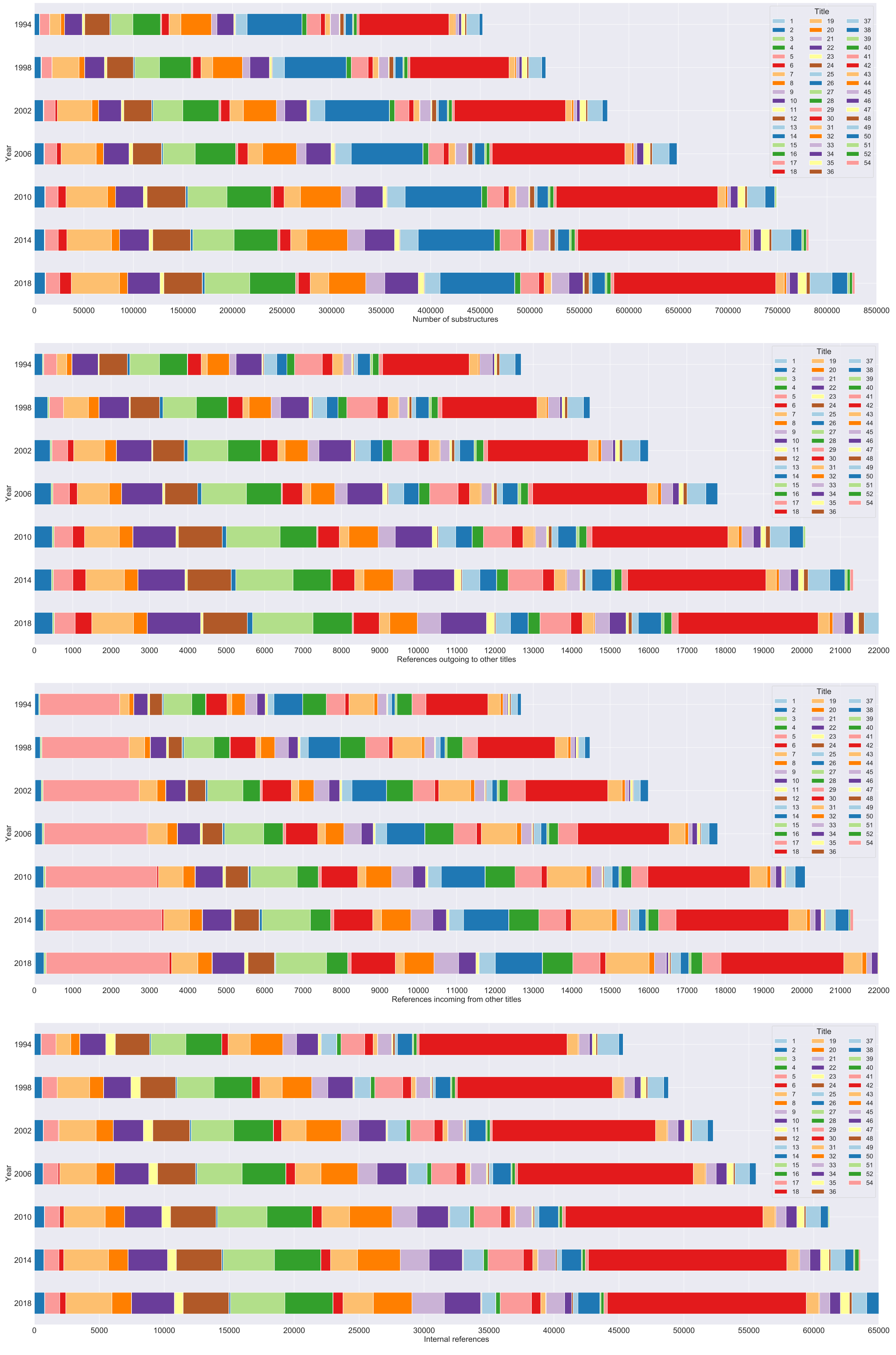

Figure 1. Federal legislation in the United States by Title (1994–2018), measured in structural units.

Graph type	Country code	Snapshot	Edge weight max.	Edge weight min.	Node size max.	Node size min.
Chapter	US	1994	182	1	986148	5010
Chapter	US	2018	343	1	1155704	5002
Chapter	DE	1994	153	1	73548	5017
Chapter	DE	2018	660	1	182847	5047
Community	US	1994	128	1	7530609	100790
Community	US	2018	356	1	8850024	156513
Community	DE	1994	179	1	2497714	100332
Community	DE	2018	393	1	3943949	152300

Table 1. Minimal and maximal values of the raw data for each graph in Figure 4 from the main paper and Figure 2. The opacity of arrows is scaled based on the edge weight extrema.

To position the nodes, we use the Fruchterman-Reingold force-directed layout algorithm as implemented in `NetworkX` with an optimal distance between nodes of $k = 2.2$ and a seed of 1234 feeded by `numpy`.

In a similar spirit, Figure 2 visualises the result of the clustering. In general, the graphic is generated like Figure 4 from the main paper, but now, each node represents a cluster. For the 1994 graphs, nodes smaller than 100000 tokens are hidden, whereas for the 2018 graphs, we hide nodes smaller than 150000 tokens. Moreover, the node size scaling is 40 times smaller than in Figure 4 from the main paper.

3.3 Main paper Figure 5

Figure 5 from the main paper provides an overview of how the US Code developed over the last 25 years in an alluvial plot. It is based on the family graph F^i for $i = \text{US}$.

We limit the number of clusters per year to 50 and condense smaller clusters into one additional cluster per year (the *miscellaneous* cluster), to focus on large and medium size clusters. Moreover, we combine multiple edges between clusters summarised in the miscellaneous cluster into one.

The clusters are ordered vertically by year. Horizontally, we sort them from left ot right by decreasing size, then force the miscellaneous cluster to the right as it condenses the smallest clusters. The clusters for one year are visualised as horizontally stacked bars. The height is fixed and the width indicates the size of the respective cluster in tokens. The horizontal width of edges indicates the weight of an edge in tokens. The scale mapping tokens to width is identical for nodes and edges in one alluvial plot but it differs across countries because the year with the most tokens in each collection is scaled to the same width. Clusters are labelled by numbers that represent the order in which our consensus clustering implementation reported the clustering results. They match the numbering in Figure 2 and should be interpreted on a nominal scale.

The cluster identifiers are hidden in Figure 5 from the main paper. Figure 3 is a copy of this figure including identifiers for detailed inspection, and Figure 4 is its analogue for Germany. HTML files in the data repository accompanying this paper describe the content of the clusters. They show the absolute size in tokens of each cluster and its size relative to the whole dataset for one year and country. The summarised elements (Chapters or laws and Books) of each cluster are listed by their path (e.g., TITLE 42-THE PUBLIC HEALTH AND WELFARE / CHAPTER 6-THE CHILDREN'S BUREAU) and their size relative to the whole cluster.

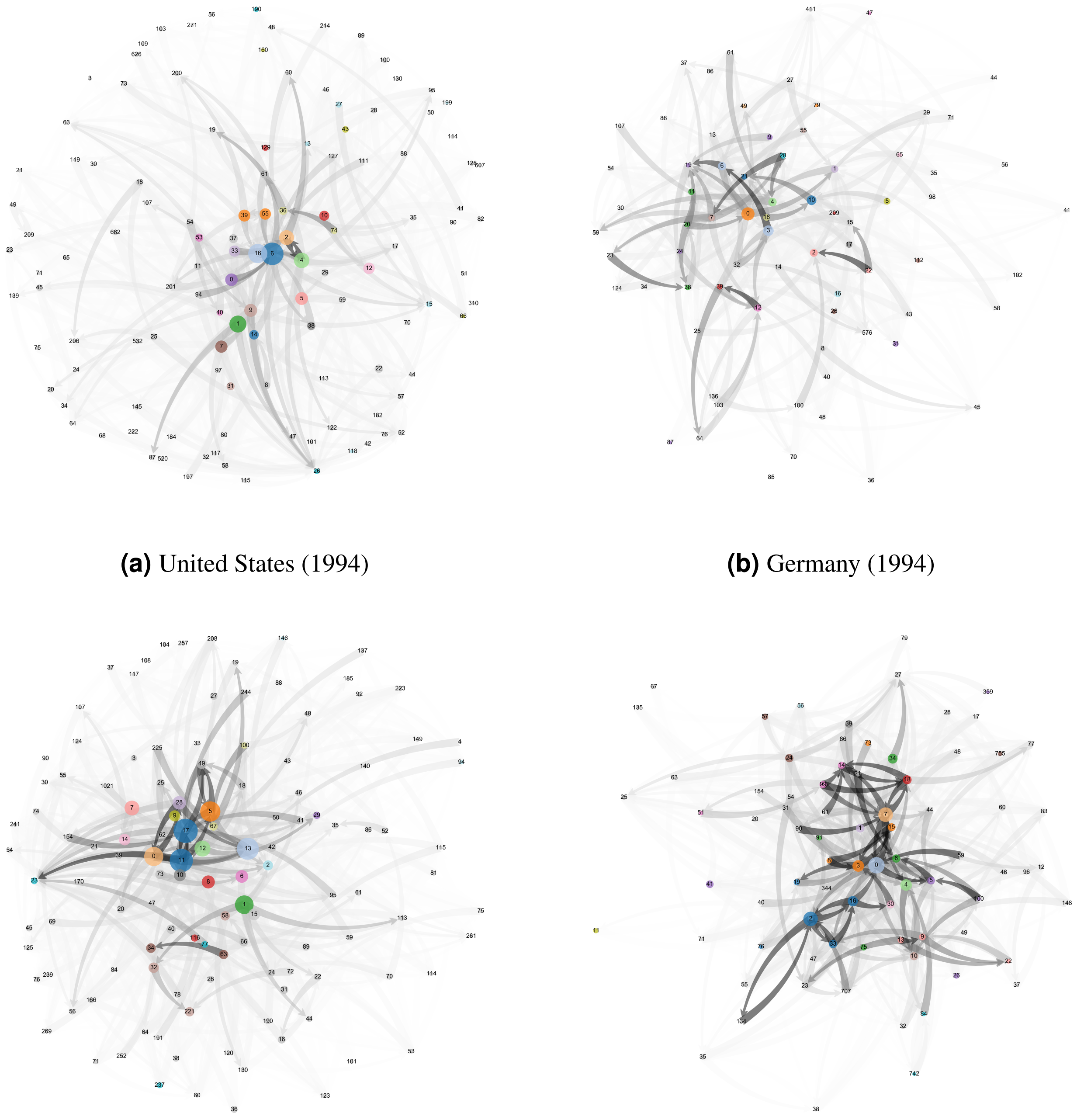

(a) United States (1994)

(b) Germany (1994)

(c) United States (2018)

(d) Germany (2018)

Figure 2. Federal legislation in the United States and Germany: quotient graphs by cluster (1994 and 2018), with arrows running between nodes indicating that text contained in one node cites text contained in another node. Node sizes indicate token counts (larger = more tokens), where only nodes with at least 100000 tokens for 1994 and 150000 tokens for 2018 are shown. Arrow colours indicate numbers of outgoing references (darker = more references). For each nation separately, nodes share the same colour if they are placed in the same cluster family, and nodes not in one of the 20 largest cluster families are coloured in grey.

Nodes and edges of the 20 largest cluster families are coloured by their relationship. Other nodes and edges are coloured in alternating greys. The miscellaneous node is coloured in light grey. Edges are plotted with opacity 0.5 and in increasing order of their weight, with the largest edges plotted last.

3.4 Main paper Figure 6

Figure 6 from the main paper and Figure 5 illustrate in scatter plots how the sizes of the cluster families evolve during the observation period. Figure 5 shows cluster family sizes for the 20 largest cluster families in each collection (United States and Germany), where the size of a cluster family is the size of the largest cluster it contains. Figure 6 from the main paper visualises a selection of the most and the least growing cluster families among the 20 largest families to facilitate interpretation. The growth of a cluster family is determined by the slope of an OLS regression for each cluster family $V^i_{F,j}$ on the cluster family sizes $|V^i_{F,j,t}|$ at times $\{\ t\ \in\ T\ \}$, where T is the observation period.

The resulting intercepts and regression slopes are shown as lines in Figure 6 from the main paper and in Figure 5. The points and lines for one cluster family have the same colours in both graphics. In Figure 5, colours are reused. To enable a mapping of lines to the legend, we order the legend according to the value expected by the OLS regression in 2018.

In Figure 5, we label cluster families by year and number of the largest cluster they contain (which also determines the overall size of the cluster family, see Definition 6 from the main paper). For the purposes of Figures 6 from the main paper, we manually assign labels to the cluster families. Here, we derive a topic from the names and token shares of Chapters, books, and laws comprising the cluster family.

Figure 6 shows the mean size of the 20 largest cluster families in relation to their slope derived by the OLS regressions as a scatter plot. We add a regression line and indicate the 95 % confidence interval using translucent bands around that regression line. Detailed statistics regarding the regression in Figures 6 from the main paper and Figure 5, and regarding the source data of Figure 6, are provided in Table 2.

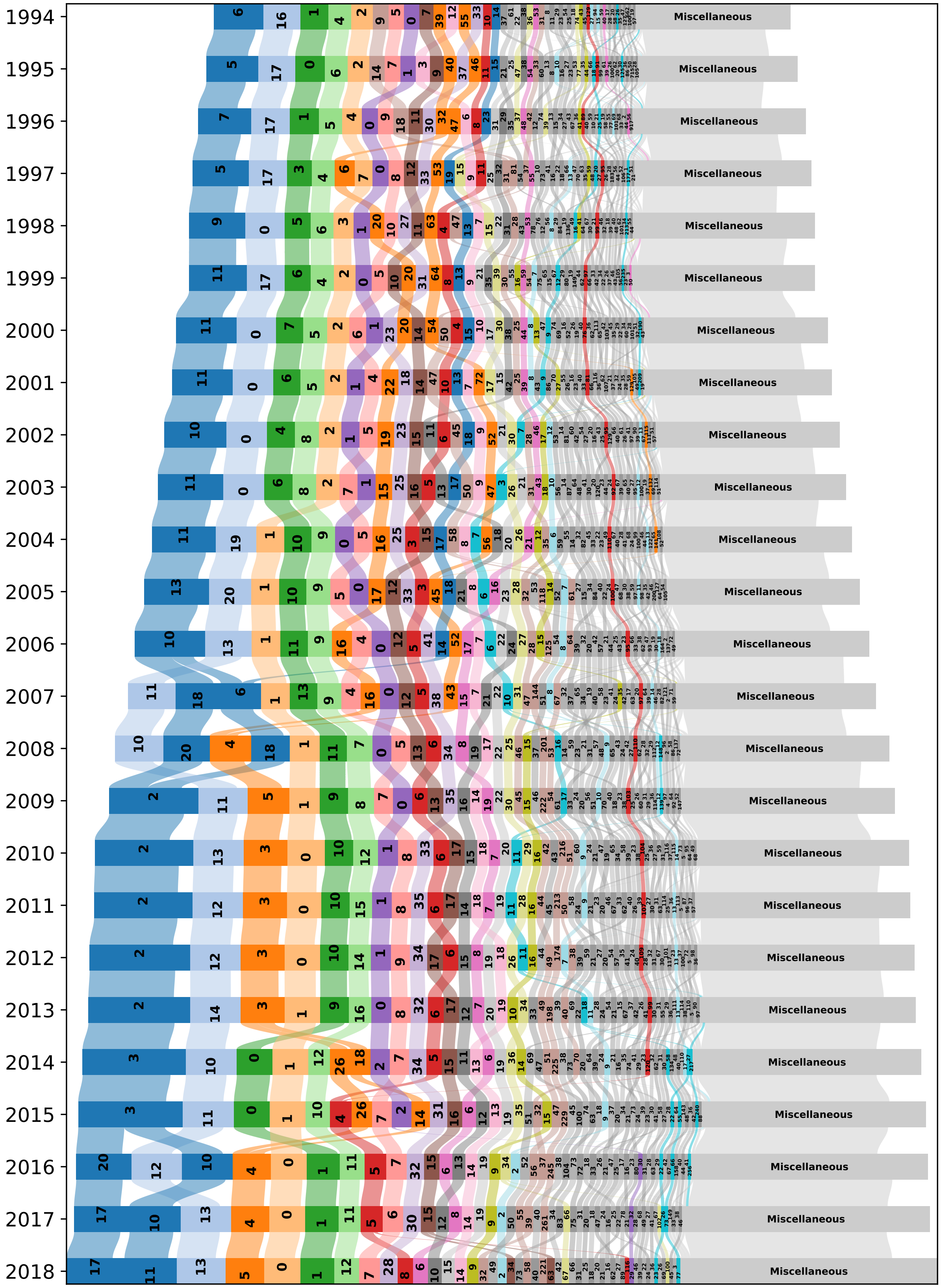

Figure 3. Federal legislation in the United States by cluster (1994–2018), with cluster numbers to enable content inspection (cf. Section 5). Each block in each year represents a cluster. Clusters are ordered from left to right by decreasing size (measured in tokens) and coloured by the cluster family to which they belong, where clusters not in one of the 20 largest cluster families are coloured in alternating greys. Small clusters are summarised in one miscellaneous cluster, which is always the rightmost cluster and coloured in light grey.

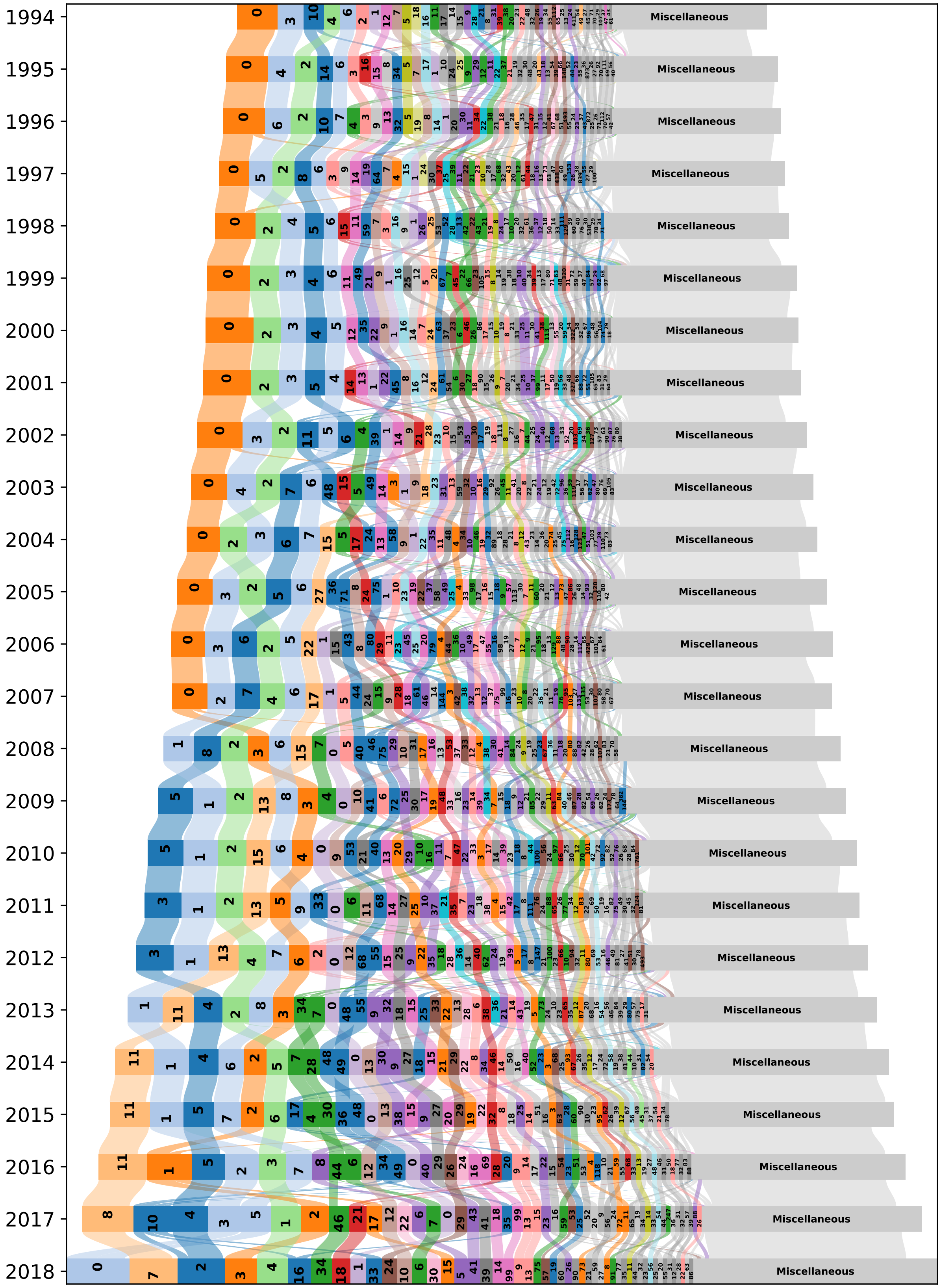

Figure 4. Federal legislation in Germany by cluster (1994–2018), with cluster numbers to enable content inspection (cf. Section 5). Each block in each year represents a cluster. Clusters are ordered from left to right by decreasing size (measured in tokens) and coloured by the cluster family to which they belong, where clusters not in one of the 20 largest cluster families are coloured in alternating greys. Small clusters are summarised in one miscellaneous cluster, which is always the rightmost cluster and coloured in light grey.

Country code	Cluster family	Slope	Intercept	P-value	Correlation coefficient (r)	r^2	Standard error	Mean value
US	3 in 2015	53488.68	1410483.92	0.00	0.99	0.98	1504.06	2052348.04
	0 in 2010	20618.14	440351.10	0.00	0.95	0.90	1423.24	687768.80
	3 in 2011	18660.65	611768.54	0.00	0.85	0.72	2427.73	835696.28
	11 in 2015	17144.95	879622.55	0.00	0.94	0.89	1246.52	1085362.00
	4 in 2015	8972.70	328139.30	0.00	0.96	0.92	555.38	435811.68
	10 in 2018	7161.79	179446.56	0.00	0.87	0.76	849.28	265388.08
	0 in 2015	6836.02	622702.52	0.00	0.69	0.48	1497.39	704734.72
	9 in 1994	6273.73	461446.27	0.00	0.71	0.50	1297.80	536731.04
	6 in 2018	5868.71	192494.97	0.00	0.92	0.86	503.29	262919.44
	7 in 2018	5833.80	372200.23	0.00	0.91	0.82	569.96	442205.88
	6 in 2005	5282.55	158014.62	0.00	0.77	0.59	913.11	221405.20
	28 in 2018	5171.32	305893.97	0.00	0.93	0.86	428.32	367949.84
	15 in 2018	5032.92	188544.60	0.00	0.93	0.87	401.49	248939.64
	2 in 2017	4913.84	248094.22	0.00	0.89	0.79	526.91	307060.24
	9 in 2018	4287.96	167592.31	0.00	0.91	0.83	404.87	219047.84
	12 in 2007	2259.43	333293.88	0.00	0.81	0.66	340.97	360407.00
	3 in 1995	964.03	272922.70	0.09	0.35	0.12	544.57	284491.00
	26 in 2012	487.62	242650.01	0.28	0.23	0.05	436.96	248501.48
	8 in 2009	470.18	566616.93	0.52	0.13	0.02	726.27	572259.04
	0 in 2013	-4522.89	454055.07	0.17	-0.28	0.08	3176.27	399780.44
DE	2 in 2018	21366.55	327039.44	0.00	0.98	0.96	928.87	583438.08
	8 in 2017	16311.05	-17159.36	0.00	0.95	0.90	1128.43	178573.28
	34 in 2018	8504.39	155890.06	0.00	0.91	0.83	803.00	257942.68
	0 in 2018	8464.01	332669.71	0.00	0.97	0.95	407.17	434237.80
	8 in 2016	6789.83	205624.20	0.00	0.97	0.94	343.14	287102.20
	15 in 2015	5047.60	97312.68	0.00	0.97	0.94	275.66	157883.88
	24 in 2018	5027.07	58539.27	0.00	0.93	0.87	407.10	118864.16
	22 in 2017	3471.46	20050.75	0.00	0.94	0.88	265.79	61708.28
	9 in 1997	2690.08	85071.52	0.00	0.92	0.84	246.24	117352.48
	0 in 2010	2576.20	74740.47	0.00	0.83	0.69	361.66	105654.88
	0 in 2000	2520.76	376433.06	0.01	0.49	0.24	928.82	406682.20
	18 in 2018	2200.20	89329.56	0.00	0.89	0.80	232.43	115731.96
	4 in 2018	2150.07	196437.59	0.00	0.62	0.39	566.59	222238.48
	2 in 2012	1985.76	159928.37	0.00	0.84	0.70	269.83	183757.48
	29 in 2016	1954.78	60064.11	0.00	0.82	0.67	289.18	83521.44
	10 in 2018	-12.21	118595.63	0.95	-0.01	0.00	209.39	118449.08
	21 in 2011	-611.70	76818.35	0.02	-0.47	0.22	237.07	69478.00
	5 in 1996	-976.76	62273.86	0.00	-0.58	0.33	287.79	50552.76
	16 in 2000	-2133.60	86460.10	0.00	-0.89	0.80	225.41	60856.92
	19 in 1996	-2923.61	59316.50	0.00	-0.72	0.52	585.81	24233.20

Table 2. Statistics regarding the OLS regressions in Figure 6 from the main paper and Figure 5. The p-value column shows a two-sided p-value for a hypothesis test whose null hypothesis is that the slope is zero, using the Wald Test with t-distribution of the test statistic.

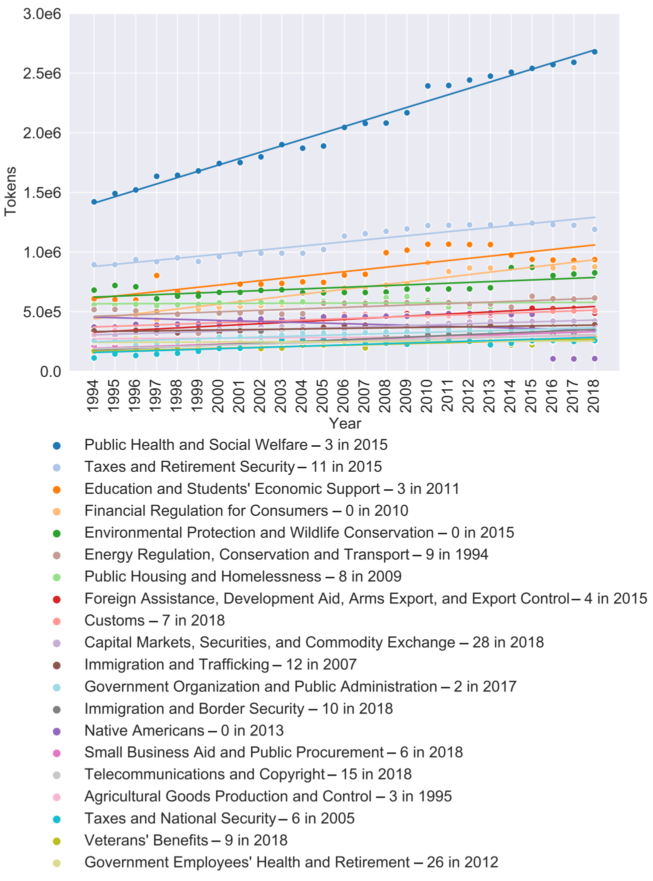

(a) United States

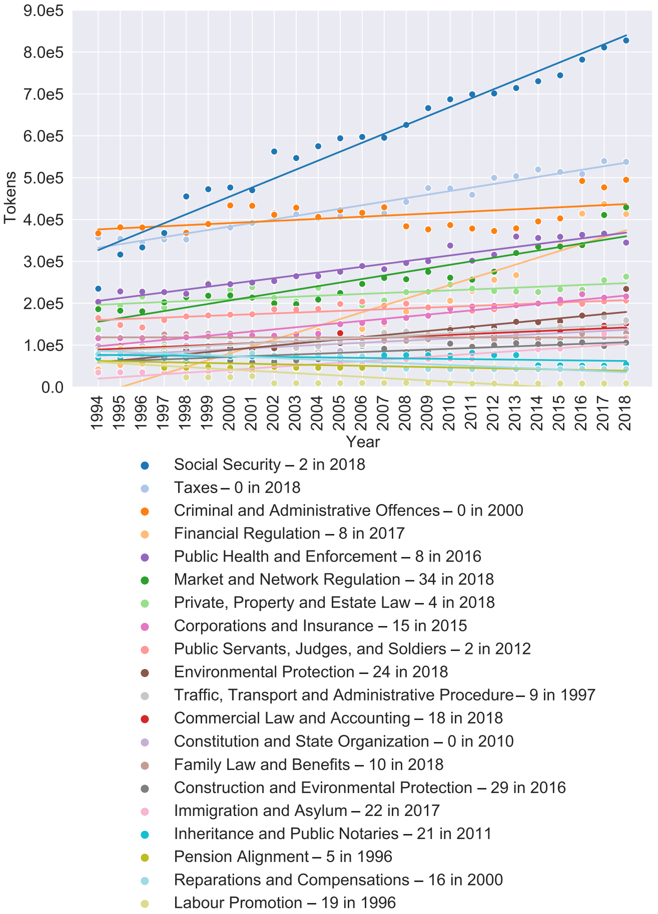

(b) Germany

Figure 5. Federal legislation in the United States and Germany: growth statistics by cluster family (1994–2018).

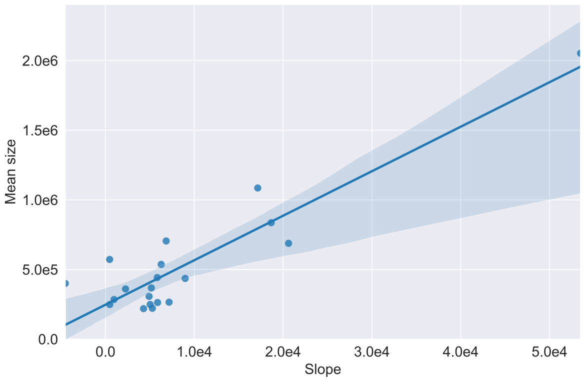

(a) United States

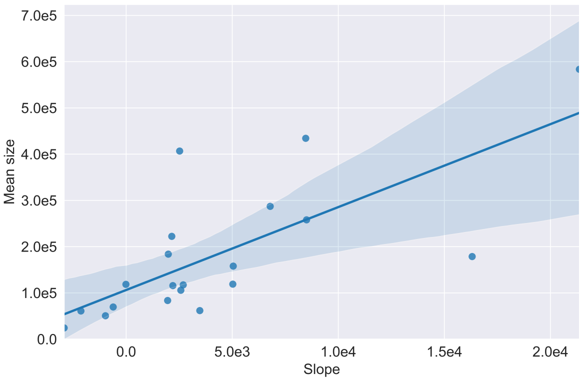

(b) Germany

Figure 6. Federal legislation in the United States and Germany: slope-to-size correlation by cluster family (1994–2018).

4 Clustering algorithm parametrisation

In the following, we analyse the performance of our clustering algorithm under different parameter choices to ensure that our results are not artefacts of our parametrisation. The statistics we report are based on the Normalized Mutual Information (NMI) and Adjusted Rand Index (ARI)—two metrics that are commonly used for pairwise comparisons of clustering results.

NMI is an information-theoretic measure expressing how much information is shared between two clusterings. It is scaled to range between 0 (not similar at all) and 1 (identical), and defined as

$$\mathrm{NMI}(X;Y) = \frac{I(X;Y)}{\sqrt{H(X)H(Y)}} ,$$

where $I(X;Y) = H(X;Y) - H(X \mid Y) - H(Y \mid X)$ is the mutual information between X and Y, $H(X;Y)$ is the joint entropy of X and Y, $H(X)$ and $H(Y)$ are the individual entropies, and $H(X \mid Y)$ and $H(Y \mid X)$ are the conditional entropies. For more information on this measure, see [2].

The ARI is variant of the Rand Index (RI) adjusted for chance. The Rand Index is based on counting how many pairs of nodes are in the same clusters or in different clusters in both clusterings. It is defined as

$$\mathrm{RI}(X;Y) = \frac{a+b}{a+b+c+d} ,$$

where a is the number of node pairs that appear in the same cluster in both clusterings, b is the number of node pairs that appear in different clusters in both clusterings, c is the number of node pairs that appear in the same cluster in X but in different clusters in Y, and d is the number of node pairs that appear in different clusters in X but in the same cluster in Y. The ARI is defined as

$$\mathrm{ARI}(X;Y) = \frac{\mathrm{RI}(X;Y) - \mathbb{E}[\mathrm{RI}(X;Y)]}{1 - \mathbb{E}[\mathrm{RI}(X;Y)]} ,$$

where $\mathbb{E}[\mathrm{RI}(X;Y)]$ is the expected RI when assuming that the X and Y partitions are constructed randomly, subject to having the original number of clusters and the original number of nodes in each cluster. While the RI ranges between 0 and 1, the ARI is bounded from above by 1 but may take negative values when the agreement between the clusterings is less than expected. Unrelated clusterings have an ARI close to 0 and identical clusterings have an ARI of 1. More information on this measure can be found in [3].

4.1 Sensitivity analysis

Figure 7 shows how the clustering results change when we alter the preferred number of clusters, with our chosen number of clusters as the baseline. As preferred numbers of clusters, we test all numbers divisible by 10 from 10 to 150 as well as the number 200. In one experiment, labelled *auto*, we let the *Infomap* algorithm choose the preferred number of clusters. Unsurprisingly, the box plots show that clusterings become more similar to our baseline clustering with 100 preferred clusters as we approach this number. At the same time, clusterings with 50 or 200 preferred clusters are already relatively similar to the baseline, with NMI values over 0.96 and ARI values over 0.7.

Note that the spread in clustering similarities is largest for comparisons of the baseline with *auto*, i.e., the clusterings in which *Infomap* chooses the preferred number of clusters. This is likely due to the jumps in clustering granularity that sometimes occur in *Infomap* due to small differences in the minimum description length of competing models with different resolutions. Avoiding these jumps is our primary motivation for specifying a preferred number of clusters.

Figure 8 shows how the compositions of cluster families change if we choose 50 or 200, rather than 100 preferred clusters, or let the algorithm determine the preferred number of clusters. As should become clear by visual inspection, the overall picture remains the same.

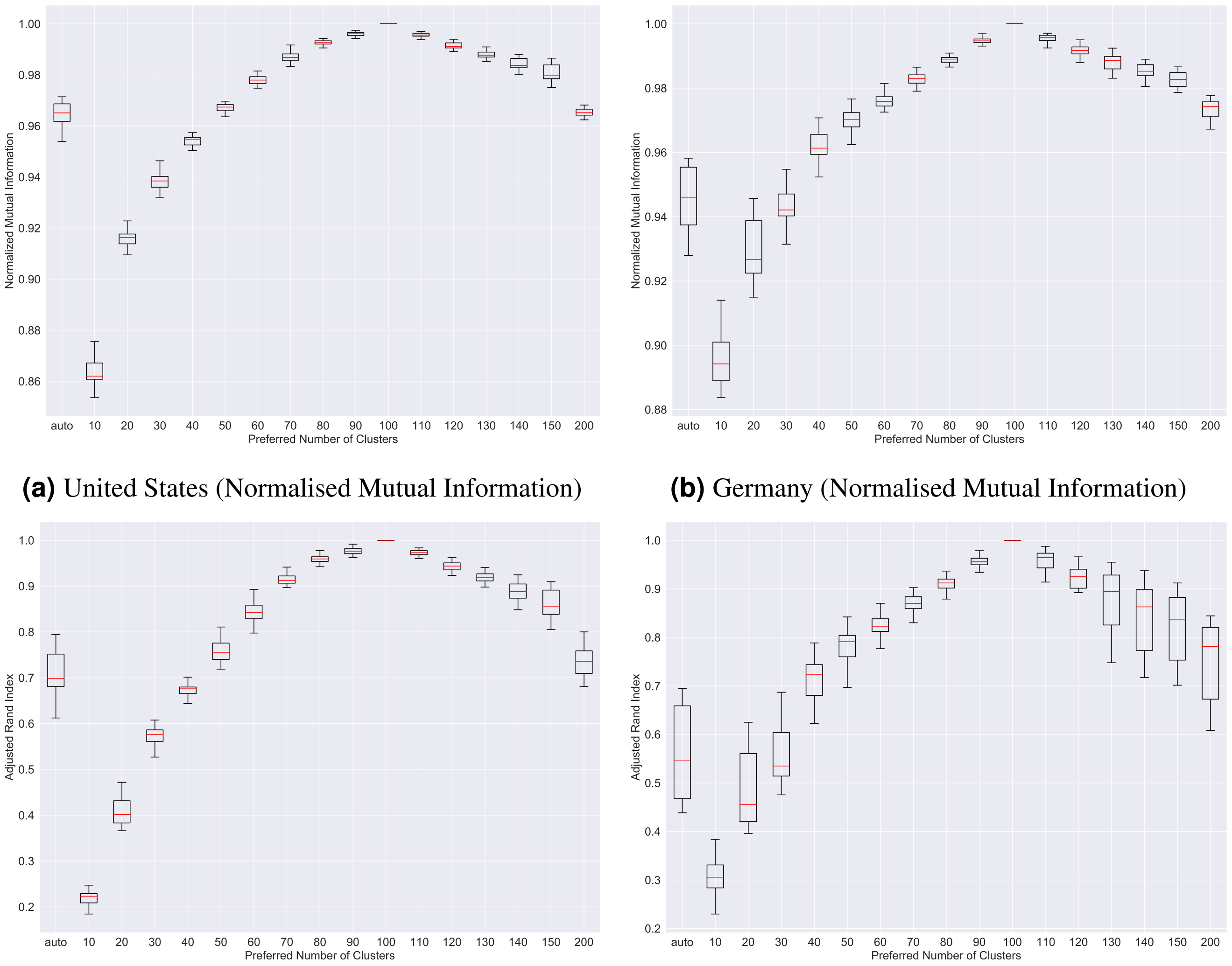

(a) United States (Normalised Mutual Information)

(b) Germany (Normalised Mutual Information)

(c) United States (Adjusted Rand Index)

(d) Germany (Adjusted Rand Index)

Figure 7. Distribution of pairwise similarities between clusterings with different preferred cluster sizes in the same year over the 25 years from 1994 to 2018. *Auto* indicates that the *Infomap* algorithm chooses the preferred number of clusters. Note that only the box plots labelled 10 through 150 are equidistant to each other on the real line. The y-coordinates of the box boundaries indicate the second and fourth quartile, while the red line indicates the median. Upper whiskers extend to the last data point less than 1.5 times the box height above the fourth quartile, while lower whiskers extend to the first data point less than 1.5 times the box height below the first quartile.

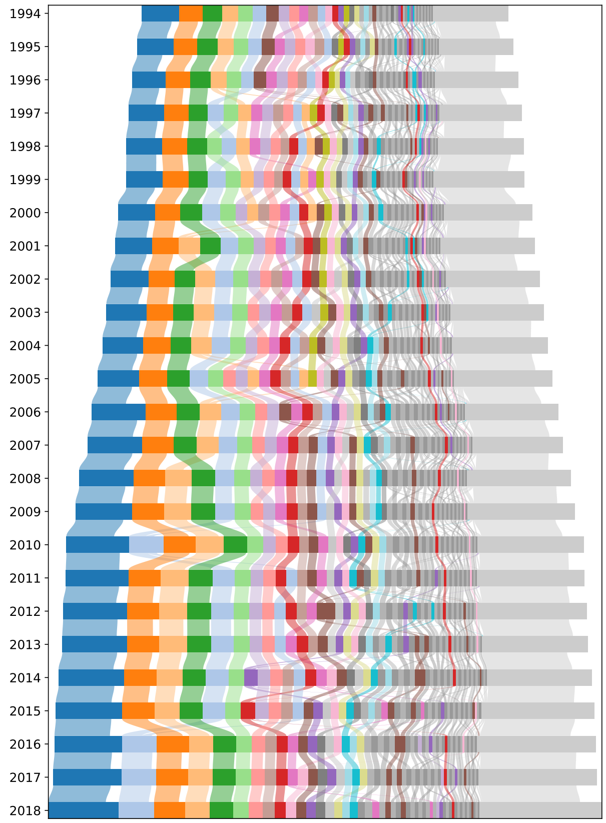

(a) 50 preferred clusters

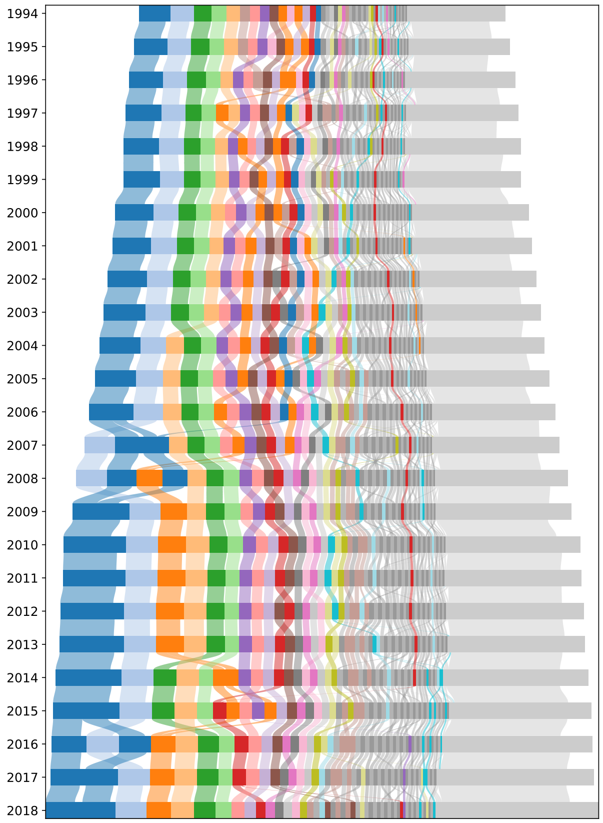

(b) 100 preferred clusters

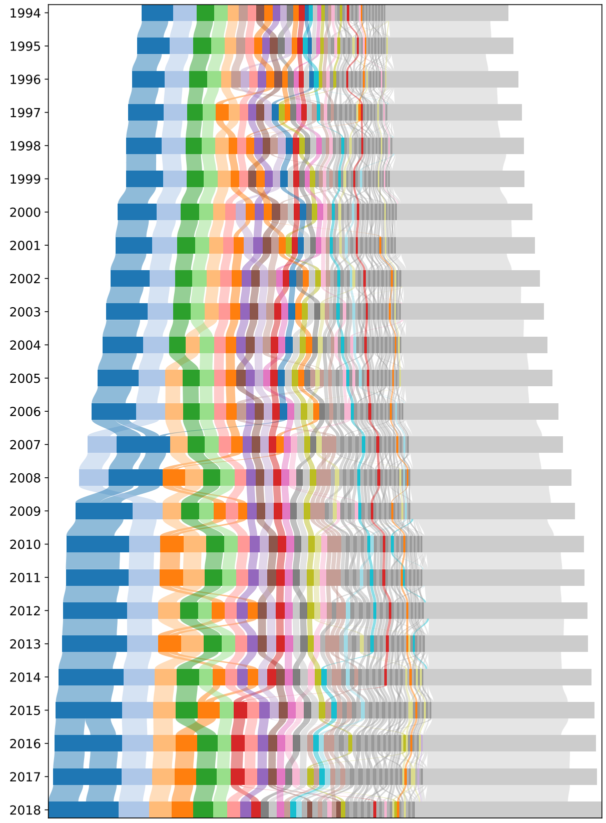

(c) 200 preferred clusters

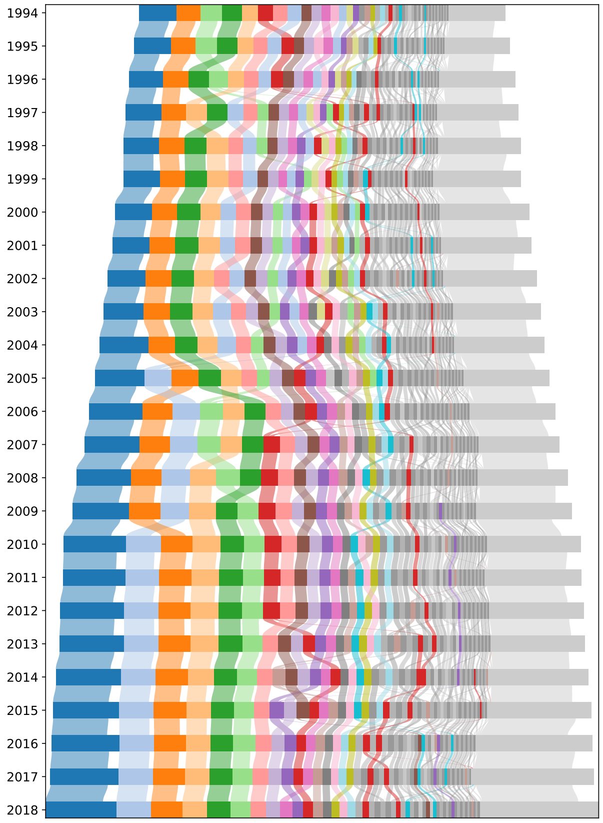

(d) Algorithm determines number of clusters

Figure 8. Federal legislation in the United States by cluster (1994–2018), depicted as in Figure 5 from the main paper, for different preferred numbers of clusters.

4.2 Robustness checks

Figure 9 shows the distribution of pairwise similarities between 100 consensus clustering results for different numbers of clusterings used in the consensus. The plots show that using a higher number of clusterings to form the consensus increases the overall similarity level and reduces the spread between the observed similarities. When choosing 1000 clusterings to form the consensus (as we do in the main paper), the consensus clusterings we obtain in different runs are almost identical.

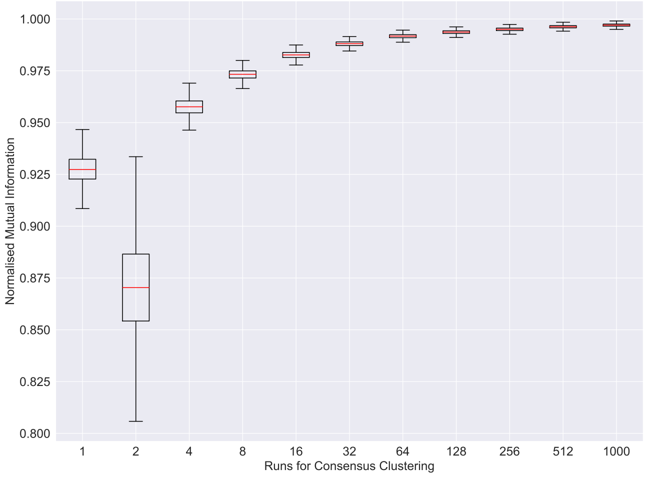

(a) United States (Normalised Mutual Information)

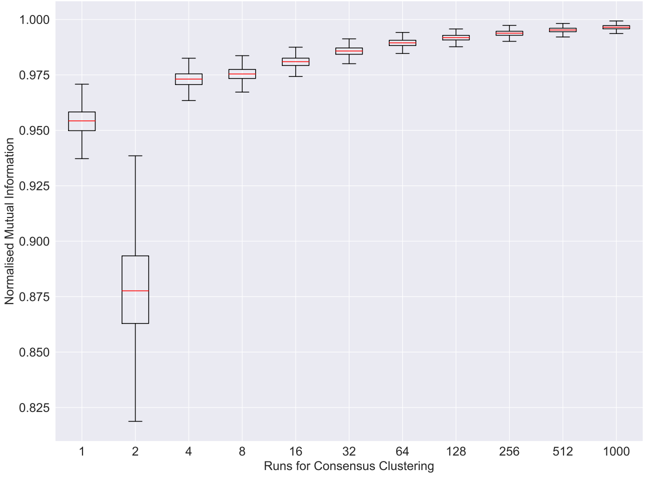

(b) Germany (Normalised Mutual Information)

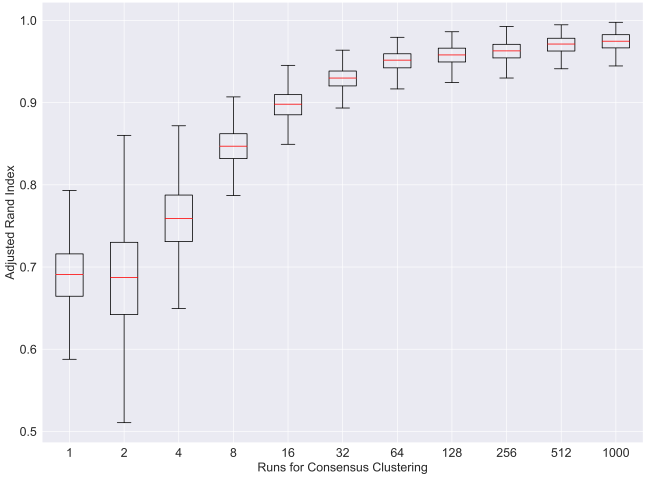

(c) United States (Adjusted Rand Index)

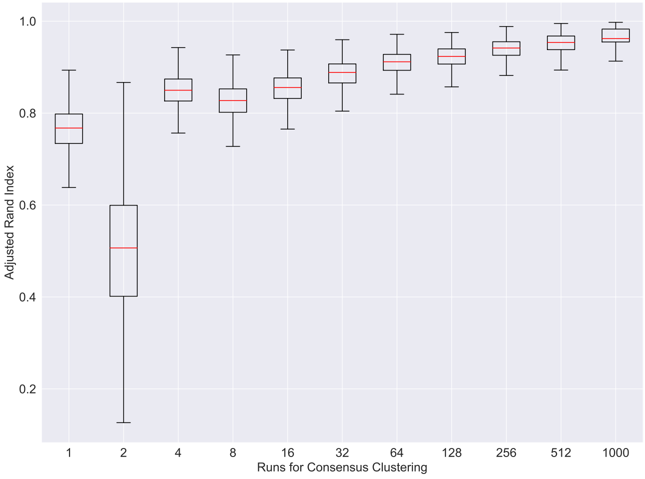

(d) Germany (Adjusted Rand Index)

Figure 9. Pairwise similarity between 100 consensus clustering results by number of clusterings used for finding the consensus (box plot interpretation as described in the caption of Figure 7).

Figure 10 shows the distribution of pairwise similarities between 100 pairs of clusterings (i.e., a total of 4950 similarities) with 100 as the preferred number of clusters. The NMI values for the United States clusterings mostly range between 0.86 and 0.94, while the NMI values for Germany mostly range between 0.84 and 0.96. The ARI values for the United States clusterings mostly range between 0.55 and 0.85 (with the majority lying between 0.65 and 0.80), while the ARI values for Germany mostly range between 0.60 and 0.90. All similarity distributions seem to shift towards the left over time, i.e., clusterings in earlier

years tend to be more similar to each other than clusterings in later years. This is likely due to the growth in complexity reported in the main paper.

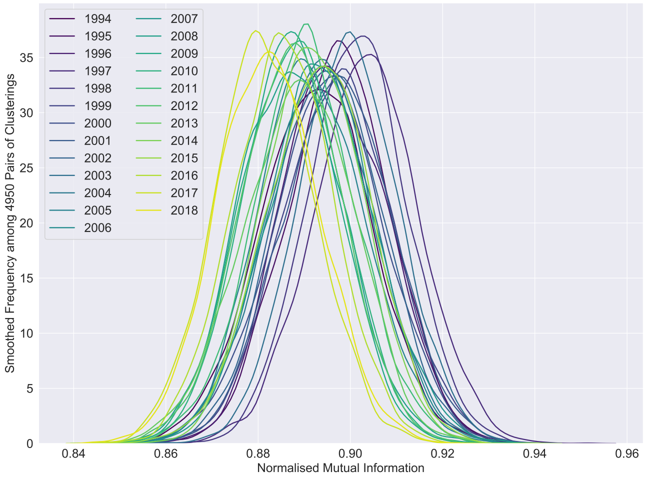

(a) United States (Normalised Mutual Information)

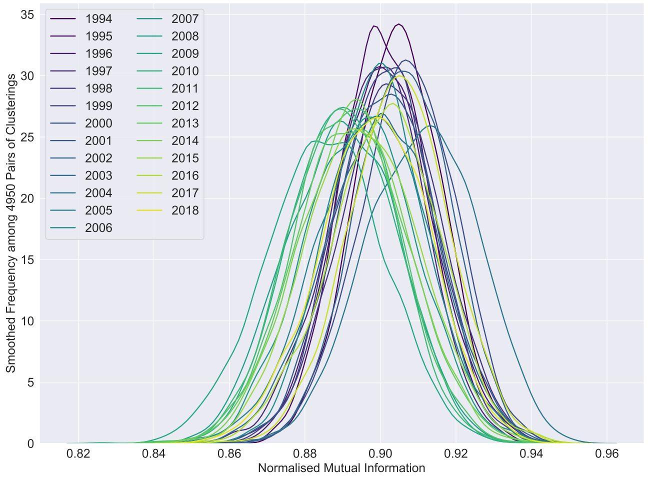

(b) Germany (Normalised Mutual Information)

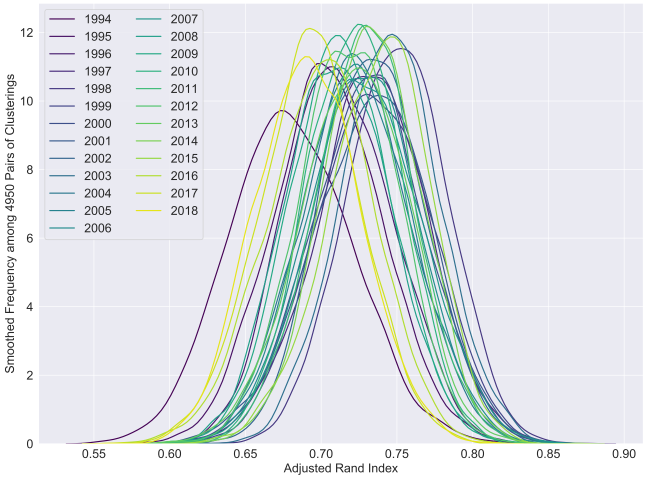

(c) United States (Adjusted Rand Index)

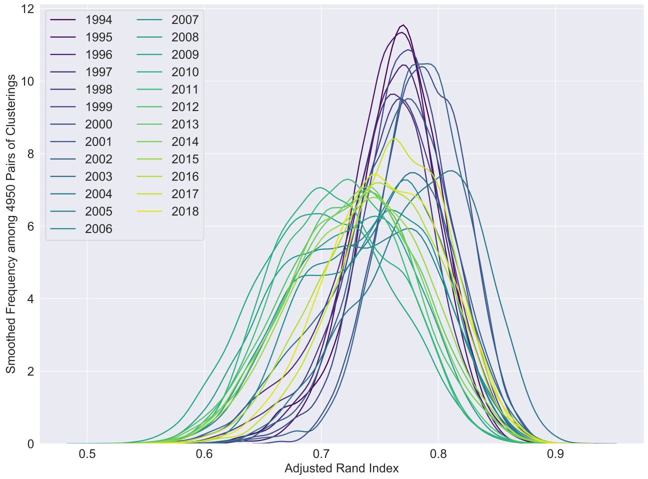

(d) Germany (Adjusted Rand Index)

Figure 10. Pairwise similarity between 100 clusterings with 100 as the preferred number of clusters, depicted as kernel-density estimates rather than frequency histograms to reduce visual clutter.

5 Cluster families

5.1 Labelling the twenty largest cluster families

To arrive at the labels of the 20 largest cluster families, we leverage our subject matter expertise, inspecting the content of the cluster families based on automatically generated summaries that show what percentage of the cluster is made up by which particular Chapter, Book, or law (measured in tokens). These summaries contain the full paths to each node, including the names of all structural elements in which it is contained in the hierarchy graph. As such, they provide just enough dimensionality reduction for humans to be able to assign the final label with confidence, and they are part of the data provided with the paper. For comparison, we also provide basic TF-IDF (term frequency-inverse document frequency; for more information, see [4]) statistics for the 20 largest cluster families as CSV files in the data repository accompanying this paper (with very little preprocessing and without stopwords removed).

Table 3 contains the Top 10 nouns according to TF-IDF for the United States cluster families depicted in Figure 6 (a) from the main paper. Table 4 shows a (reformatted) excerpt from the summary of the largest cluster family in the United States as used in our manual labelling process. The full lists of our labels for the 20 largest clusters are reproduced in Tables 5 and 6.

	**2015-3**	**2015-11**	**2011-3**	**2010-0**	**2015-4**	**2009-8**	**1995-3**
1	pesticide	multiemployer	student	depository	cuba	mortgagee	acreage
2	secretary	year	teachers	thrift	hiv	mortgagor	cotton
3	state	taxpayer	youth	conservator	states	homeownership	wheat
4	medicare	plan	secretary	sipc (*)	pakistan	secretary	crop
5	drug	purposes	school	bank	mtcr (**)	housing	quota
6	services	amount	state	depositor	afghanistan	mortgages	peanuts
7	physician	dividend	teacher	banking	nato	mortgage	sugar
8	pediatric	corporation	childhood	institution	hungary	dwelling	upland
9	health	income	agency	board	democracy	homebuyers	tobacco
10	vaccine	distributee	education	corporation	israel	paint	milk

Table 3. Top 10 nouns for the 7 cluster families depicted in Figure 6 (a) from the main paper, labelled by leading cluster (Year-Cluster Identifier). Nouns referring to structural elements of legal texts (e.g., title, section, subsection, paragraph) are excluded. (*) Securities Investor Protection Corporation. (**) Missile Technology Control Regime.

5.2 Inspecting the *miscellaneous* clusters

Recall that although we use 100 as the preferred number of clusters, we end up with more than 100 clusters due to the presence of nodes without any incoming or outgoing references (*singletons*) and our use of consensus clustering. To reduce visual clutter, in Figure 5 from the main paper, we limit the number of clusters drawn per year to 50, summarising the remaining clusters in one additional miscellaneous cluster.

In both the United States and Germany, in all years, the miscellaneous cluster contains mostly singletons or near-singletons corresponding to small Chapters or laws that are largely self-contained. Its composition remains fairly stable over time (i.e., nodes in the miscellaneous cluster seldom get pulled into a different cluster), and its growth is primarily driven by the addition of new, relatively independent

Chapters or laws. Since its contents are very diverse, the growth of the miscellaneous cluster could be interpreted as an indicator that our legal corpora grow not only in volume but also in diversity.

To illustrate that the clusters we summarise in the miscellaneous clusters have little impact on our results, Figure 11 juxtaposes analogues of Figure 5 from the main paper that summarise only clusters behind the 500th largest cluster in a miscellaneous cluster with their original counterparts that summarise all clusters behind the 50th cluster, where clusters are sorted in decreasing order of their size.

**Leading Cluster**	**Percentage**	**Chapter Path**
1994-6	81.35	TITLE 42-THE PUBLIC HEALTH AND WELFARE / CHAPTER 6-THE CHILDREN'S BUREAU
	6.19	TITLE 42-THE PUBLIC HEALTH AND WELFARE / CHAPTER 34-ECONOMIC OPPORTUNITY PROGRAM
	3.30	TITLE 45-RAILROADS / CHAPTER 9-RETIREMENT OF RAILROAD EMPLOYEES
1994-14	53.21	TITLE 21-FOOD AND DRUGS / CHAPTER 8-NARCOTIC FARMS
	14.77	TITLE 7-AGRICULTURE / CHAPTER 6-INSECTICIDES AND ENVIRONMENTAL PESTICIDE CONTROL
	10.91	TITLE 15-COMMERCE AND TRADE / CHAPTER 47-CONSUMER PRODUCT SAFETY
1998-9	49.30	TITLE 42-THE PUBLIC HEALTH AND WELFARE / CHAPTER 7-SOCIAL SECURITY
	32.88	TITLE 42-THE PUBLIC HEALTH AND WELFARE / CHAPTER 6A-PUBLIC HEALTH SERVICE
	4.21	TITLE 42-THE PUBLIC HEALTH AND WELFARE / CHAPTER 35-PROGRAMS FOR OLDER AMERICANS
1998-13	50.87	TITLE 21-FOOD AND DRUGS / CHAPTER 9-FEDERAL FOOD, DRUG, AND COSMETIC ACT
	14.81	TITLE 7-AGRICULTURE / CHAPTER 6-INSECTICIDES AND ENVIRONMENTAL PESTICIDE CONTROL
	8.33	TITLE 15-COMMERCE AND TRADE / CHAPTER 47-CONSUMER PRODUCT SAFETY
2002-10	48.65	TITLE 42-THE PUBLIC HEALTH AND WELFARE / CHAPTER 7-SOCIAL SECURITY
	35.10	TITLE 42-THE PUBLIC HEALTH AND WELFARE / CHAPTER 6A-PUBLIC HEALTH SERVICE
	3.69	TITLE 7-AGRICULTURE / CHAPTER 51-FOOD STAMP PROGRAM
2002-18	54.65	TITLE 21-FOOD AND DRUGS / CHAPTER 9-FEDERAL FOOD, DRUG, AND COSMETIC ACT
	13.77	TITLE 7-AGRICULTURE / CHAPTER 6-INSECTICIDES AND ENVIRONMENTAL PESTICIDE CONTROL
	7.82	TITLE 15-COMMERCE AND TRADE / CHAPTER 47-CONSUMER PRODUCT SAFETY
2006-10	50.39	TITLE 42-THE PUBLIC HEALTH AND WELFARE / CHAPTER 7-SOCIAL SECURITY
	34.42	TITLE 42-THE PUBLIC HEALTH AND WELFARE / CHAPTER 6A-PUBLIC HEALTH SERVICE
	3.67	TITLE 42-THE PUBLIC HEALTH AND WELFARE / CHAPTER 35-PROGRAMS FOR OLDER AMERICANS
2006-14	53.84	TITLE 21-FOOD AND DRUGS / CHAPTER 9-FEDERAL FOOD, DRUG, AND COSMETIC ACT
	13.28	TITLE 7-AGRICULTURE / CHAPTER 6-INSECTICIDES AND ENVIRONMENTAL PESTICIDE CONTROL
	6.79	TITLE 15-COMMERCE AND TRADE / CHAPTER 47-CONSUMER PRODUCT SAFETY
2010-2	43.74	TITLE 42-THE PUBLIC HEALTH AND WELFARE / CHAPTER 7-SOCIAL SECURITY
	29.89	TITLE 42-THE PUBLIC HEALTH AND WELFARE / CHAPTER 6A-PUBLIC HEALTH SERVICE
	10.85	TITLE 21-FOOD AND DRUGS / CHAPTER 9-FEDERAL FOOD, DRUG, AND COSMETIC ACT
2014-3	42.90	TITLE 42-THE PUBLIC HEALTH AND WELFARE / CHAPTER 7-SOCIAL SECURITY
	28.56	TITLE 42-THE PUBLIC HEALTH AND WELFARE / CHAPTER 6A-PUBLIC HEALTH SERVICE
	12.75	TITLE 21-FOOD AND DRUGS / CHAPTER 9-FEDERAL FOOD, DRUG, AND COSMETIC ACT
2018-17	82.07	TITLE 42-THE PUBLIC HEALTH AND WELFARE / CHAPTER 7-SOCIAL SECURITY
	5.05	TITLE 7-AGRICULTURE / CHAPTER 51-SUPPLEMENTAL NUTRITION ASSISTANCE PROGRAM
	4.47	TITLE 42-THE PUBLIC HEALTH AND WELFARE / CHAPTER 35-PROGRAMS FOR OLDER AMERICANS
2018-11	59.96	TITLE 42-THE PUBLIC HEALTH AND WELFARE / CHAPTER 6A-PUBLIC HEALTH SERVICE
	27.72	TITLE 21-FOOD AND DRUGS / CHAPTER 9-FEDERAL FOOD, DRUG, AND COSMETIC ACT
	4.26	TITLE 7-AGRICULTURE / CHAPTER 6-INSECTICIDES AND ENVIRONMENTAL PESTICIDE CONTROL

Table 4. Top 3 contents of clusters in family 0 (leading cluster: 2015-3), labelled "Public Health and Social Welfare", in four-year intervals from 1994 to 2018.

	Leading Cluster	Color	Label
1	2015-3		Public Health and Social Welfare
2	2010-0		Financial Regulation for Consumers
3	2011-3		Education and Students' Economic Support
4	2015-11		Taxes and Retirement Security
5	2015-4		Foreign Assistance, Development Aid, Arms Export, and Export Control
6	2018-10		Immigration and Border Security
7	2015-0		Environmental Protection and Wildlife Conservation
8	1994-9		Energy Regulation, Conservation, and Transport
9	2018-6		Small Business Aid and Public Procurement
10	2018-7		Customs
11	2005-6		Taxes and National Security
12	2018-28		Capital Markets, Securities, and Commodity Exchange
13	2018-15		Telecommunications and Copyright
14	2017-2		Government Organization and Public Administration
15	2018-9		Veterans' Benefits
16	2007-12		Immigration and Trafficking
17	1995-3		Agricultural Goods Production and Control
18	2012-26		Government Employees' Health and Retirement
19	2009-8		Public Housing and Homelessness
20	2013-0		Native Americans

Table 5. Labels assigned to the 20 largest cluster families in the United States, ordered by regression slope (cf. Table 2).

	Leading Cluster	Color	Label
1	2018-2		Social Security
2	2017-8		Financial Regulation
3	2018-34		Market and Network Regulation
4	2018-0		Taxes
5	2016-8		Public Health and Enforcement
6	2015-15		Corporations and Insurance
7	2018-24		Environmental Protection
8	2017-22		Immigration and Asylum
9	1997-9		Traffic, Transport and Administrative Procedure
10	2010-0		Constitution and State Organization
11	2000-0		Criminal and Administrative Offences
12	2018-18		Commercial Law and Accounting
13	2018-4		Private Law, Property Law, and Estate Law
14	2012-2		Public Servants, Judges, and Soldiers
15	2016-29		Construction and Environmental Protection
16	2018-10		Family Law and Benefits
17	2011-21		Inheritance and Public Notaries
18	1996-5		Pension Alignment
19	2000-16		Reparations and Compensations
20	1996-19		Labour Promotion

Table 6. Labels assigned to the 20 largest cluster families in Germany, ordered by regression slope (cf. Table 2).

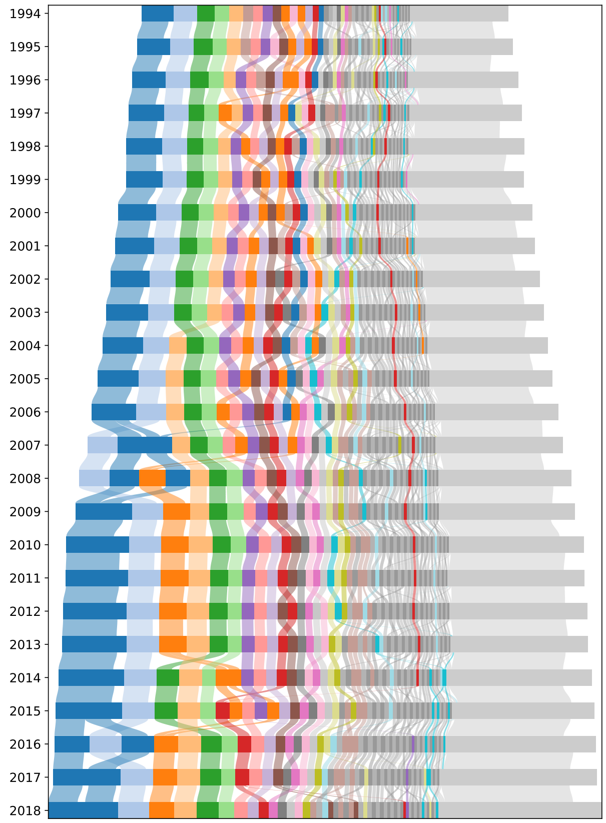

(a) United States (50 + 1 clusters drawn per year)

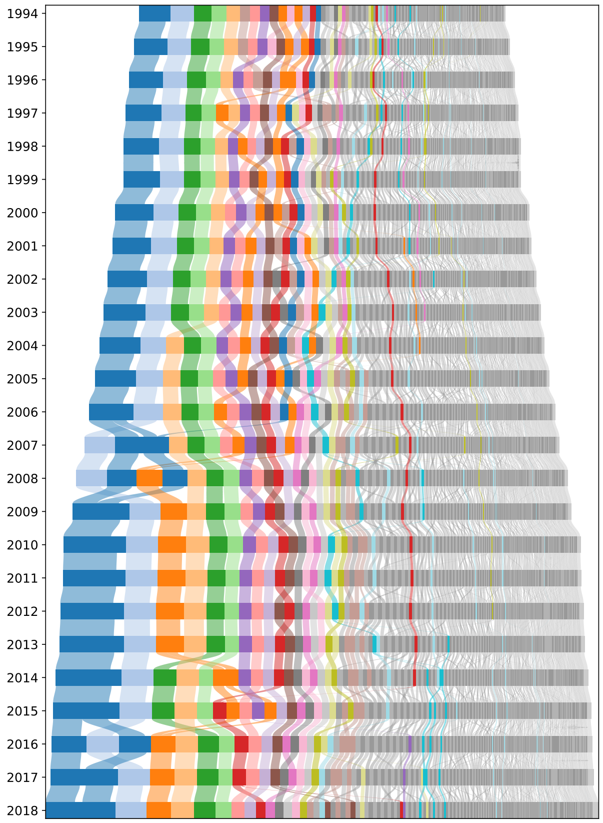

(b) United States (500 + 1 clusters drawn per year)

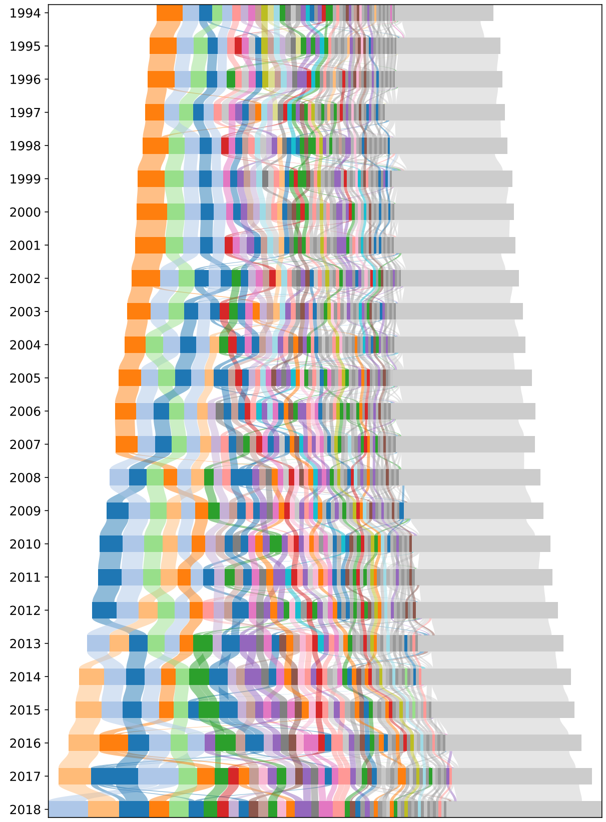

(c) Germany (50 + 1 clusters drawn per year)

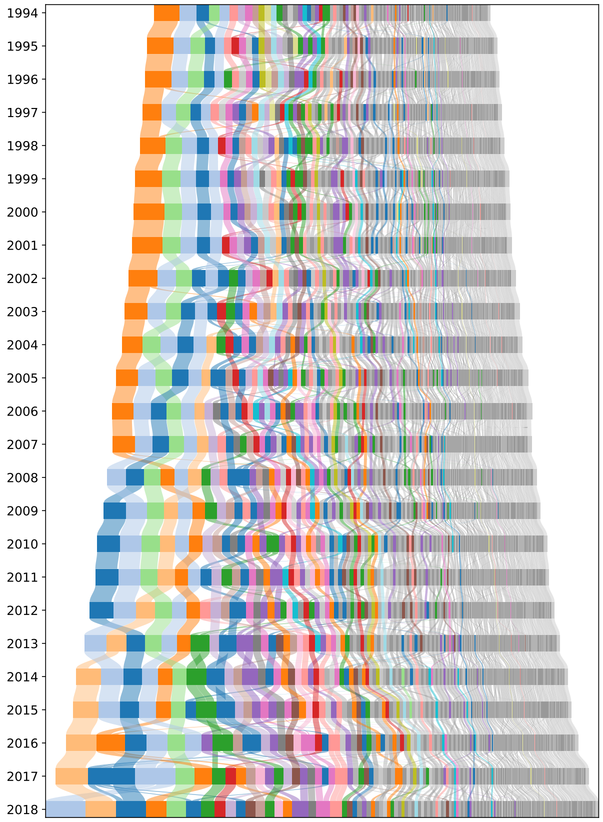

(d) Germany (500 + 1 clusters drawn per year)

Figure 11. Federal legislation in the United States and Germany by cluster (1994–2018), depicted as in Figure 5 from the main paper, with different thresholds for summarising small clusters in one miscellaneous cluster.

References

1. Bommarito, M. J. & Katz, D. M. A mathematical approach to the study of the united states code. *Physica A* **389**, 4195–4200 (2010).

2. Strehl, A. & Ghosh, J. Cluster ensembles—a knowledge reuse framework for combining multiple partitions. *Journal of machine learning research* **3**, 583–617 (2002).

3. Hubert, L. & Arabie, P. Comparing partitions. *Journal of classification* **2**, 193–218 (1985).

4. Schütze, H., Manning, C. D. & Raghavan, P. *Introduction to information retrieval* (Cambridge University Press Cambridge, 2008).
